# Functional mapping and annotation of genetic associations with FUMA

**DOI:** 10.1038/s41467-017-01261-5

**Published:** 2017-11-28

**Authors:** Kyoko Watanabe, Erdogan Taskesen, Arjen van Bochoven, Danielle Posthuma

**Affiliations:** 1 Department of Complex Trait Genetics, Center for Neurogenomics and Cognitive Research, VU University Amsterdam, Amsterdam, 1081 HV The Netherlands; 2 VU University Medical Center (VUMC), Alzheimercentrum, Amsterdam, 1081 HV The Netherlands; 3 Faculty of Science, VU University Amsterdam, Amsterdam, 1081 HV The Netherlands; 4 Department of Clinical Genetics, VU University Medical Center, Amsterdam Neuroscience, Amsterdam, 1081 HV The Netherlands

## Abstract

A main challenge in genome-wide association studies (GWAS) is to pinpoint possible causal variants. Results from GWAS typically do not directly translate into causal variants because the majority of hits are in non-coding or intergenic regions, and the presence of linkage disequilibrium leads to effects being statistically spread out across multiple variants. Post-GWAS annotation facilitates the selection of most likely causal variant(s). Multiple resources are available for post-GWAS annotation, yet these can be time consuming and do not provide integrated visual aids for data interpretation. We, therefore, develop FUMA: an integrative web-based platform using information from multiple biological resources to facilitate functional annotation of GWAS results, gene prioritization and interactive visualization. FUMA accommodates positional, expression quantitative trait loci (eQTL) and chromatin interaction mappings, and provides gene-based, pathway and tissue enrichment results. FUMA results directly aid in generating hypotheses that are testable in functional experiments aimed at proving causal relations.

## Abstract (web summary)

Prioritizing genetic variants is a major challenge in genome-wide association studies. Here, the authors develop FUMA, a web-based bioinformatics tool that uses a combination of positional, eQTL and chromatin interaction mapping to prioritize likely causal variants and genes.

## Introduction

In the past decade, more than 2500 genome-wide association studies (GWAS) have identified thousands of genetic loci for hundreds of traits^1^. The past 3 years have seen an explosive increase in GWAS sample sizes^2–4^, and these are expected to increase even further to 0.5–1 million in the next year and beyond^5^. These well-powered GWAS will not only lead to more reliable results but also to an increase in the number of detected disease-associated genetic loci. To benefit from these results, it is crucial to translate genetic loci into actionable variants that can guide functional genomics experimentation and drug target testing^6^. However, since the majority of GWAS hits are located in non-coding or intergenic regions^7^, direct inference from significantly associated single-nucleotide polymorphisms (SNPs) rarely yields functional variants. More commonly, GWAS hits span a genomic region (“GWAS risk loci”) that is characterized by multiple correlated SNPs, and may cover multiple closely located genes. Some of these genes may be relevant to the disease, while others are not, yet due to the correlated nature of closely located genetic variants, distinguishing relevant from non-relevant genes is often not possible based on association *P*-values alone. Pinpointing the most likely relevant, causal genes and variants requires integrating available information about regional linkage disequilibrium (LD) patterns and functional consequences of correlated SNPs, such as deleteriousness of variants, but also their effects on gene expression as well as their role in chromatin interaction sites. Ideally, functional inferences obtained from different repositories are integrated, and annotated SNP effects are interpreted in the broader context of genes and molecular pathways. For example, consider a genomic risk locus with one lead SNP associated with an increased risk for a disease, and several dozen other SNPs in LD with the lead SNP that also show a low association *P*-value, spanning multiple genes. If none of these tested SNPs and none of the other (not tested but known) SNPs in LD with the lead SNP are known to have a functional consequence (i.e., altering expression of a gene, affecting a binding site or violating the protein structure), no causal gene can be indicated. However, if one or several of the SNPs are known to affect the function of one of the genes in the area, but not the other genes, then that single gene has a higher probability of being functionally related to the disease. Pinpointing which and how genes are affected by SNPs associated with a trait is crucial in increasing our insight into the biological mechanisms underlying that trait. Interpreting SNP-trait associations requires adding functional information from several resources and repositories such as, e.g., the Genotype-Tissue Expression (GTEx)^8^, Encyclopedia of DNA Elements (ENCODE)^9^, Roadmap Epigenomics Project^10^, or chromatin interaction information^11^.

In practice, the extraction and interpretation of the relevant biological information from available repositories is not always straightforward, and can be time consuming as well as error prone. We have, therefore, developed FUMA, which functionally annotates GWAS findings and prioritizes the most likely causal SNPs and genes using information from 18 biological data repositories and tools. Gene prioritization is based on a combination of positional mapping, expression quantitative trait loci (eQTL) mapping and chromatin interaction mapping. Results are visualized to facilitate quick insight into the implicated molecular functions. FUMA is available as an online tool at http://fuma.ctglab.nl, where users can customize settings to for example only use exonic SNPs for annotation, or only use SNPs that are eQTLs in specific tissues for the annotation based on expression data. As input, FUMA requires GWAS summary statistics and outputs include multiple tables and figures containing extensive information on, e.g., functionality of SNPs in genomic risk loci, including protein-altering consequences, gene-expression influences, open-chromatin states as well as three-dimensional (3D) chromatin interactions. The online tool includes interactive figures that can be used to explore associations in more depth and aids, e.g., in identifying multiple lines of evidence pointing to the same prioritized gene, or in connecting hits in several genes via biological pathways.

## Results

### Overview of FUMA web application

FUMA incorporates 18 biological data repositories and tools to process GWAS summary statistics and provide a variety of annotations (Supplementary Table 1). To accomplish this task, FUMA consists of two separate processes described in detail below.

**Fig. 1:**
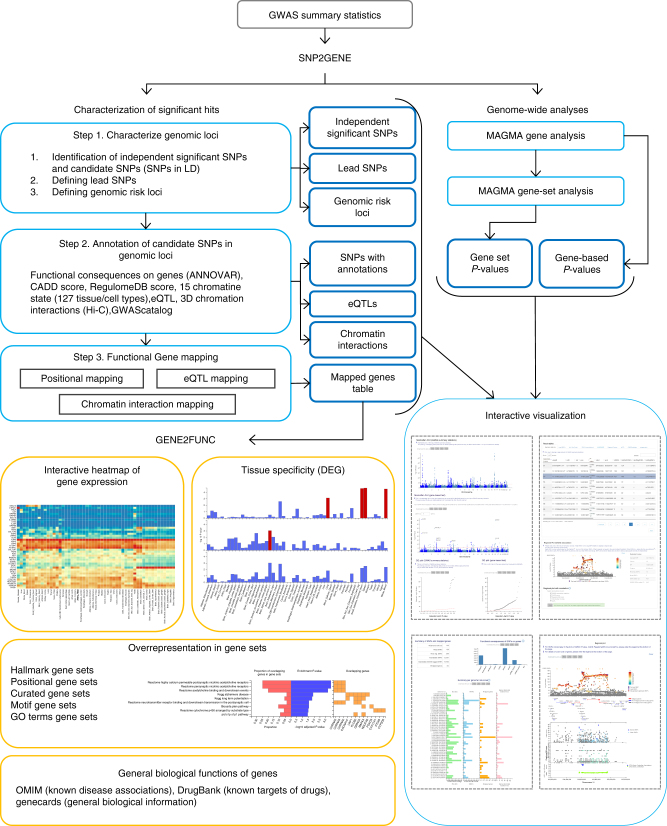
Overview of FUMA. FUMA includes two core processes, SNP2GENE and GENE2FUNC. The input is GWAS summary statistics. SNP2GENE prioritizes functional SNPs and genes, outputs tables (blue boxes), and creates Manhattan, quantile–quantile (QQ) and interactive regional plots (box at right bottom). GENE2FUNC provides four outputs; a gene expression heatmap, enrichment of differentially expressed gene (DEG) sets in a certain tissue compared to all other tissue types, overrepresentation of gene sets, and links to external biological information of input genes. All results are downloadable as text files or high-resolution images

The core function of FUMA is the SNP2GENE process (Fig.1) in which SNPs are annotated with their biological functionality and mapped to genes based on positional, eQTL and chromatin interaction information of SNPs. First, based on the provided summary statistics (input format is available in Supplementary Note 1), independent significant SNPs and their surrounding genomic loci are identified by FUMA depending on LD structure, and define lead SNPs and genomic risk loci (Methods). Independent significant SNPs and SNPs that are in LD with the independent significant SNPs are then annotated for functional consequences on gene functions (based on Ensembl genes (build 85) using ANNOVAR^12^), deleteriousness score (CADD score^13^), potential regulatory functions (RegulomeDB score^14^ and 15-core chromatin state predicted by ChromHMM^15^ for 127 tissue/cell types^9,10^), effects on gene expression using eQTLs of various tissue types and 3D structure of chromatin interactions with Hi-C data (Methods). In addition, independent significant SNPs and correlated SNPs are also linked to the GWAS catalog^1^ to provide insight into previously reported associations of the SNPs in the risk loci with a variety of phenotypes.

Functionally annotated SNPs are subsequently mapped to genes based on functional consequences on genes by (i) physical position on the genome (positional mapping), (ii) eQTL associations (eQTL mapping), and (iii) 3D chromatin interactions (chromatin interaction mapping). Gene mapping can be controlled by setting several parameters (Supplementary Table 2) that allow to in- or exclude specific functional categories of SNPs (Supplementary Fig. 1). Positional mapping is used to map SNPs based on being physically located inside a gene using a default of 10 kb windows, yet custom windows around a gene can be set by the user. Users can select to only use SNPs that have specified functional consequences, such as coding or splicing SNPs, to limit the positional mapping to functionally relevant SNPs. Thus, by selecting to exclude intronic SNPs from the positional mapping function, genes that contain only intronic SNPs in LD of independent significant SNPs will not be prioritized by FUMA. eQTL mapping is used to map SNPs to genes which they show a significant eQTL association with (i.e., the expression of that gene is associated with allelic variation at the SNP). eQTL mapping uses information from 4 data repositories (GTEx^8^, Blood eQTL browser^16^, BIOS QTL browser^17^ and BRAINEAC^18^), and is currently based on *cis*-eQTLs which can map SNPs to genes up to 1 Mb apart. Users can select tissue/cell types that are relevant to the phenotype of interest, and eQTLs can be filtered either by nominal *P*-value or FDR provided by the original data sources (Methods and Supplementary Note 2). Chromatin interaction mapping is used to map SNPs to genes when there is a significant chromatin interaction between the disease-associated regions and nearby or distant genes. Chromatin interaction mapping can involve long-range interactions as it does not have a distance boundary as in eQTL mapping. FUMA currently contains Hi-C data of 14 tissue types and seven cell lines from the study of Schmitt et al.^11^, yet new chromatin interaction data will be added when it becomes available and FUMA also allows users to upload their own chromatin interaction matrices, which is not limited to Hi-C, but also accommodates ChIA-PET, 5C or Capture Hi-C data (Methods and Supplementary Note 3). Since chromatin interactions are often defined in a certain resolution (as a genomic region), such as 40 kb, an interacting region may span multiple genes. To further prioritize candidate genes from chromatin interaction mapping, information on tissue/cell type specific enhancer and promoter regions from the Roadmap Epigenomics Project^10^ can be optionally integrated with interacting regions to filters SNPs and target genes (see Methods for details).

For each of these three mapping strategies, additional filtering of SNPs based on functional annotations (i.e., CADD, RegulomeDB, and 15-core chromatin state) is optionally available (Methods and Supplementary Table 2). For example, setting a CADD score threshold will cause FUMA to use only highly deleterious SNPs or filtering SNPs by RegulomeDB score or open chromatin state prioritizes SNPs which are likely to affect regulatory elements per one of the mapping strategies.

The three mapping strategies (positional, eQTL and chromatin interaction mapping) result in a set of prioritized genes, based on the GWAS input and specific user-defined filter settings. Both eQTL and chromatin interaction mapping may lead to prioritized genes that are not necessarily themselves located inside a genomic risk locus, although they are linked to SNPs within a genomic risk locus. The combination of positional mapping of deleterious coding SNPs, eQTL mapping, and chromatin interaction mapping across (relevant) tissue types may reveal multiple lines of evidence pointing towards the same genes and enables to prioritize genes that are highly likely involved in the trait of interest.

To obtain insight into putative biological mechanisms of prioritized genes, the GENE2FUNC process annotates these genes in biological context (Fig. 1; see Methods for details). Specifically, biological information for each input gene is provided to gain insight into previously associated diseases as well as drug targets by mapping OMIM^19^ ID and DrugBank^20^ ID. Tissue specific expression patterns based on GTEx v6 RNA-seq data^8^ for each gene are visualized as an interactive heatmap. Beside the single gene level analyses, overrepresentation in sets of differentially expressed genes (DEG; sets of genes which are more (or less) expressed in a specific tissue compared to other tissue types) for each of 53 tissue types based on GTEx v6 RNA-seq data^8^ is also provided to identify tissue specificity of prioritized genes (Methods; Supplementary Table 3). Enrichment of prioritized genes in biological pathways and functional categories is tested using the hypergeometric test against gene sets obtained from MsigDB^21^ and WikiPathways^22^. The proportions of overlapping genes, enrichment *P*-value and which input genes are overlapping with the tested gene sets are visualized in plots as well as tables, which provides quick overview of the shared biological functions of prioritized genes.

The results of SNP2GENE and GENE2FUNC processes are displayed as either interactive tables or plots on the web application. Additionally, tables are downloadable as plain text files (Supplementary Note 1) and plots are downloadable as high-quality images in several formats (PNG, JPEG, PDF, and SVG).

### FUMA covers various features of existing tools

As a variety of bioinformatics tools have been developed to obtain insights in GWAS results^23–25^, we compared the list of features available in FUMA with the features available in other tools, and describe these further below (Table 1).

**Table 1:** Feature comparison of bioinformatics tools and data sources

Tools	Format	GWAS summary statistics	LD	Functional consequences on genes	Regulatory elements	eQTLs	3D chromatin interactions	Prioritize SNPs	Map SNPs to genes	Gene expression	Pathways and gene sets	Prioritize genes	Visualization
*LD calculation*													
PLINK	St	x	x										
*Variant annotations*													
ANNOVAR	St			x	x			x	x				
VEP	St			x	x			x	x				
SCAN	Web		x			x		x		x			
ReglomeDB	Web				x	x		x					x
HaploReg	Web		x		x	x		x					
*Gene-based test/Gene-set analyses*													
VEGAS	St	x							x			x	
MAGMA	St	x							x		x	x	
Pascal	St	x							x		x	x	
MAGENTA	St	x							x		x	x	
INRICH	St	x							x		x		
DEPICT	St	x							x		x	x	
*Visualization tools*													
LocusZoom	St/Web	x											x
LocusTrack	St/Web	x			x								x
3D genome browser	Web						x						x
*FUMA*													
	Web	x	x	x	x	x	x	x	x	x	x	x	x

St Standalone software, Web Web-based application

LD calculation is the first step to characterize risk loci of GWAS by computing population specific LD structure, so called clumping which identifies independent significant SNPs and defines the genomic risk loci. PLINK^26^ is the most widely used software for this task which takes GWAS summary statistics (requiring a reference panel) or genotype data as input. In FUMA, this task is automated by using pairwise LD (*r* ^2^) of SNPs in the reference panel (1000 genomes project phase 3^27^) pre-computed by PLINK, resulting in a list of independent significant SNPs, lead SNPs and genomic risk loci based on the GWAS input file. FUMA also adds SNPs to the identified risk loci that do not have a *P*-value (i.e., they were not available in the GWAS input file), but that are LD proxies of the identified lead SNPs, as these SNPs might be causally relevant. Alternatively, users can pre-compute lead SNPs or risk loci and upload these to FUMA.

Variant Annotation is required to obtain information on biological consequences of SNPs in the risk loci. There are several tools such as ANNOVAR^12^ and VEP^28^ which annotate functional consequences on genes, and variant scores such as deleteriousness and phylogenetic conservations (extensive review is available in Hou and Zhang^29^). Particularly for non-coding SNPs, SCAN^30^, RegulomeDB^14^ and HaploReg^31^ annotate regulatory information, such as eQTLs, enhancer/promoter regions, and transcription factor binding sites (see Tak and Farnham^32^ for extensive overview). Although SCAN and HaploReg correct for LD, the input of the tools mentioned above is a list of SNPs of interest which does not take genetic associations into account and thus requires pre-processing of GWAS results by the user. FUMA performs annotation of SNPs that are in LD of independent significant SNPs in a single flow, and does not require additional data preformatting.

Gene-based test/gene-set analyses are methods that enable to summarize SNP associations at the gene level and associate the set of genes to biological pathways. For instance, VEGAS performs permutation based simulation^33,34^, MAGMA employs multiple linear regression^35^ and Pascal computes sum and maximum of chi-squared statistics^36^ to obtain gene-based *P*-values. Additionally, there are several tools that perform not only gene-based test but also gene-set analyses using full distribution of genetic associations (e.g., MAGMA^35^, MAGENTA^37^, INRICH^38^, and DEPICT^39^). FUMA implements MAGMA gene-based analysis and gene-set analysis on the full GWAS input data. In addition, genes prioritized by SNP2GENE or by the user are also tested for overrepresentation in various gene sets in GENE2FUNC process.

Visualization is one of the essential features that allows (quick) insights into the GWAS results, e.g., summarizing annotated information of SNPs and genes. LocusZoom is one of the most widely used visualization tool for GWAS results which plots LD structure of a risk locus, gene locations as well as SNP association values^40^. LocusTrack is an extension of LocusZoom which also plots additional information together such as Chip-seq and chromatin state^41^. 3D Genome Browser is a recently developed web application which contains comprehensive 3D chromatin interaction datasets such as Hi-C and ChIA-PET^42^, though it does not integrate with GWAS summary statistics. These tools are primarily focused on visualization of a subset of functionally relevant data sources. FUMA integrates results from multiple lines of evidence and provides interactive visualization of results, facilitating rapid interpretation.

The current lack of a single platform that integrates all possible resources for post-GWAS annotation hampers our understanding of GWAS results, as different GWAS studies may use a different selection of queried resources rendering their post-GWAS interpretation incomplete and difficult to compare. FUMA provides a central place for a wide variety of post-GWAS annotation strategies and to our knowledge is the most versatile tool in doing so.

### Application to GWAS of body mass index

**Fig. 2:**
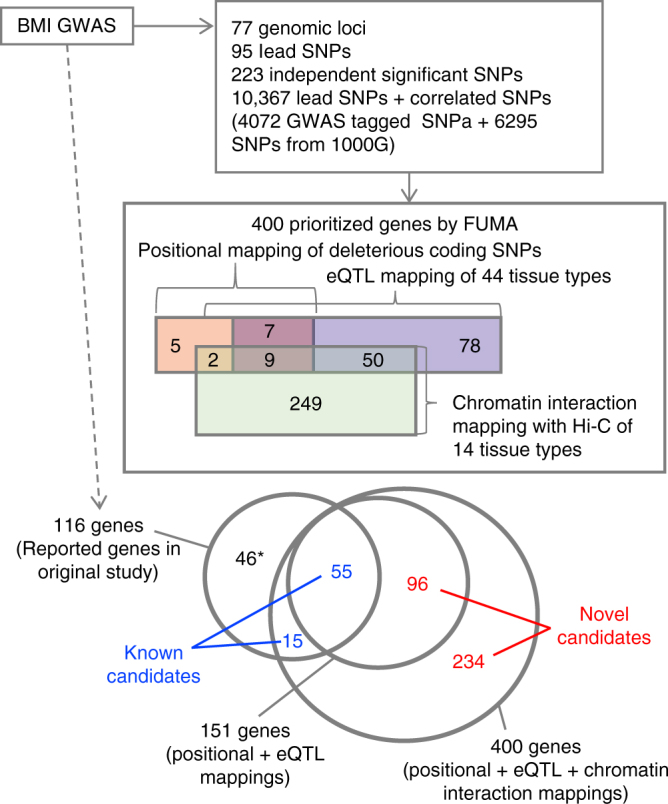
Overview of prioritized genes from BMI GWAS by FUMA. Starting from the BMI GWAS summary statistics, boxes represent results of the SNP2GENE process. The annotated SNPs include all independent lead SNPs and SNPs which are in LD with these lead SNPs. Prioritized genes are divided into three categories; genes that are implicated by deleterious coding SNPs (colored pink), by eQTLs for these genes (colored blue), or by chromatin interactions (colored green). The prioritized genes are further categorized into previously reported genes (blue) and novel genes (red) prioritized genes by FUMA. ^*^These genes were not prioritized by FUMA since they do not have either deleterious coding SNPs, eQTLs or chromatin interactions, although they are located within GWAS risk loci

**Fig. 3:**
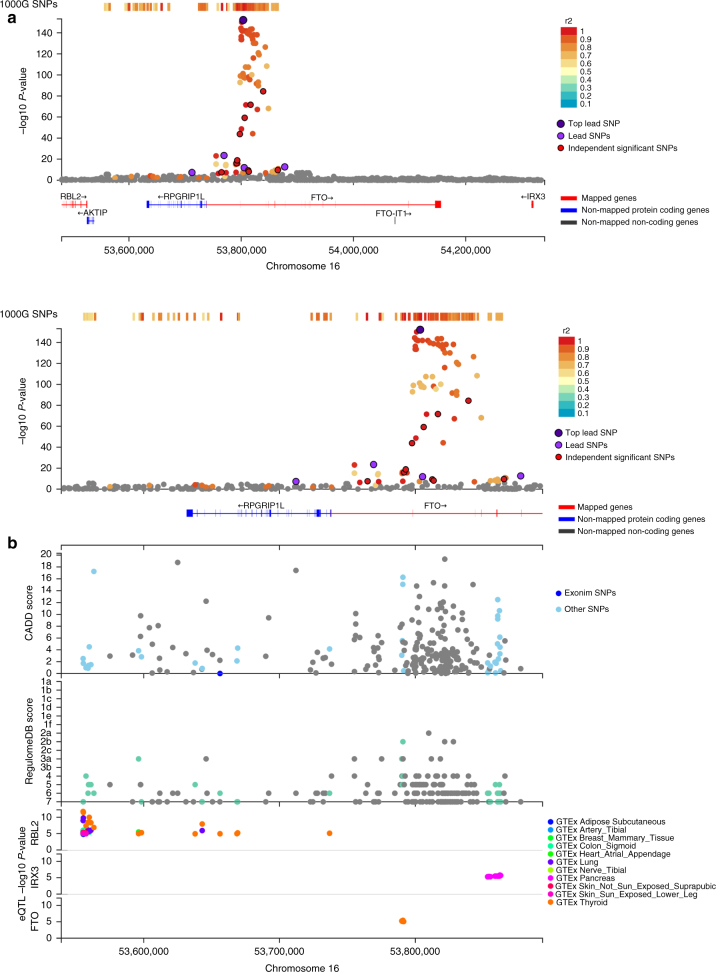
Regional plot of the locus 16q.12.2 of BMI GWAS. **a** Extended region of the FTO locus, which includes prioritized genes *RBL2* and *IRX3*. Genes prioritized by FUMA are highlighted in red. **b** Zoomed in regional plot of FTO locus with, from the top, GWAS *P*-value (SNPs are colored based on *r* ^2^), CADD score, RequlomeDB score and eQTL *P*-value. Non-GWAS-tagged SNPs are shown at the top of the plot as rectangles since they do not have a *P*-value from the GWAS, but they are in LD with the lead SNP. eQTLs are plotted per gene and colored based on tissue types. In the plots of CADD score, RegulomeDB score and eQTLs, SNPs which are not mapped to any gene are colored gray

To validate the utility of FUMA, we applied it to summary statistics of the most recent GWAS for body mass index (BMI; 236,231 individuals)^43^. FUMA identified 95 lead SNPs (from 223 independent significant SNPs) across 77 genomic risk loci (Fig. 2 and Supplementary Data 1–3), in accordance with the original study. We first conducted positional mapping of deleterious coding SNPs and eQTL mapping (Methods) which prioritized 151 unique genes; 23 genes with deleterious coding SNPs (positional mapping), and 144 genes with eQTLs that potentially alter expression of these genes (eQTL mapping) including 16 genes that had both deleterious coding SNPs and eQTLs (Supplementary Data 4). The 151 genes consist of 55 genes that were also reported in the original study^43^ and 96 novel genes implicated by FUMA, including 45 genes which are located outside the risk loci. These novel candidates have shared biological functions with the 55 previously known candidate genes such as “metabolism of carbohydrate”, “metabolism of lipid and lipoprotein”, “immune system”, and “calcium signaling” (Supplementary Data 5). In addition, FUMA results showed that, although several genomic loci for BMI included multiple prioritized genes, a single gene was prioritized in 22 out of 43 loci which contain at least one prioritized gene (Supplementary Fig. 2), suggesting that these 22 genes have a high probability of being the causal gene in that region. The 22 “highly likely causal genes” include several well-known genes for BMI such as *NEGR1*, *TOMM40*, and *TMEM18*. The strongest GWAS association signal for BMI was on 16q.12.2 where three genes were prioritized; *FTO*, *RBL2*, and *IRX3* (Fig. 3). These three genes were only prioritized by eQTL mapping as the positional mapping showed no deleterious coding SNPs located in these genes. The original study^43^ only mentioned *FTO*, because the associated SNPs were located in this gene, however none of the associated SNPs have a potential direct affect such as coding SNPs on *FTO*. Two of the genes prioritized by FUMA (*RBL2* and *IRX3*) are physically located outside the genomic locus and are missed when using conventional approaches that prioritize genes located in the locus of interest based on LD around the top SNP. Although the *IRX3* gene was not reported in the original study^43^, recent functional work has indeed validated this as the causal gene whose expression is affected by SNPs in the 16q.12.2 locus^44^.

**Fig. 4:**
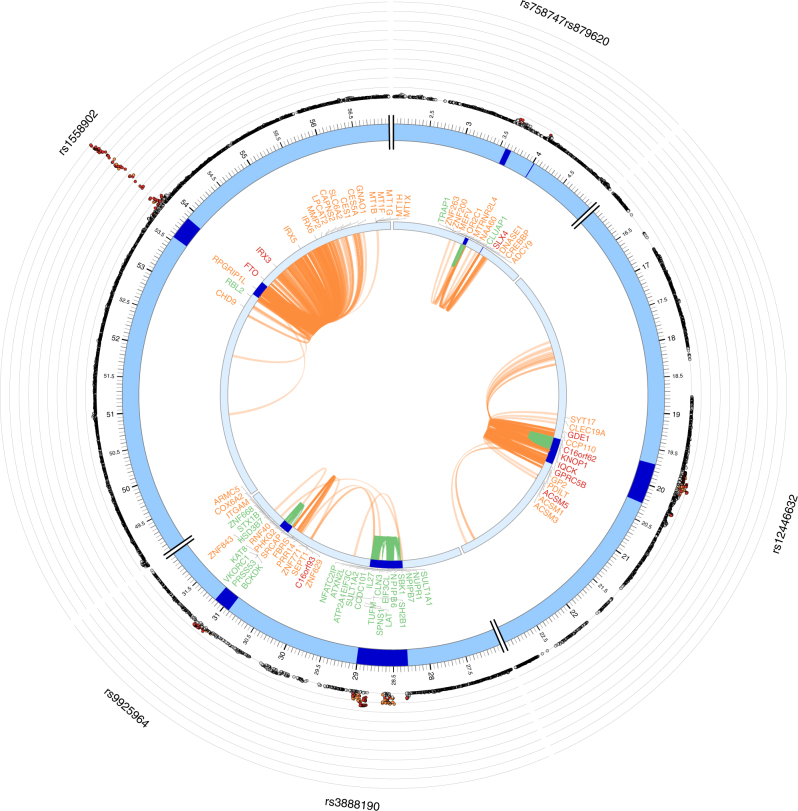
Chromatin interactions and eQTLs of BMI risk loci on chr. 16. The most outer layer is the Manhattan plot displaying SNPs with *P*-value < 0.05. Candidate SNPs are colored based on the highest *r* ^2^ to one of the independent significant loci (red: *r* ^2^ > 0.8, orange: *r* ^2^ > 0.6). Other SNPs are colored in gray. rsID of top SNPs per locus are labeled. The outer circle is the chromosome coordinate and genomic risk loci are highlighted in blue. Genes mapped by either Hi-C or eQTLs are shown on the inner circle. Genes mapped by Hi-C, eQTLs are colored orange and green, respectively. Genes mapped by both are colored red. Chromatin interaction and eQTLs are shown as links colored orange and green respectively

We then performed chromatin interaction mapping using Hi-C data of 14 tissue types (Methods). FUMA prioritized 310 genes (Supplementary Data 4), of which 61 genes are overlapping with the genes prioritized by positional and/or eQTL mappings and 232 genes are located outside of the genomic risk loci (Fig. 2). That resulted in a total of 400 prioritized genes by combining three mapping strategies including 330 novel candidates which were not reported in the original study (Table 2 and Supplementary Data 4). These novel candidates further supported shared biological functions with previously reported known genes, such as lipid and lipoprotein metabolism, homeostatic process and various metabolic pathways, with a greater number of genes compared to the mappings without Hi-C data (Supplementary Data 5). Out of 400 prioritized genes, 59 genes are mapped by both eQTLs and chromatin interactions including *IRX3* on the 16q.12.2 locus (Fig. 4), which further supports the hypothesis that these genes are involved in the risk of BMI. Of the 48 loci that contained at least one prioritized gene from positional and eQTL mappings, chromatin interaction mapping identified candidate genes in additional 18 loci (Supplementary Fig. 2), including loci mapped to known genes associated with BMI such as *MC4R*, *FOXO3*, and *ADCY9*. The 400 prioritized genes showed enrichment in 9 GO terms, such as “response to zinc ion” and “oligopeptide binding” overlapping with multiple metallothionein and glutathione S-Transferase genes whose association with obesity risk has been reported^45,46^ (Supplementary Data 6).

**Table 2:** Summary of FUMA application to three GWAS summary statistics

GWAS	Risk loci	Reported genes in the original study	Positional mapping	eQTL mapping	Chromatin interaction mapping	Total^*^	Genes located outside the risk loci	Novel candidates	Loci contain prioritized genes
BMI	77	117	23	144	310	400	263	263	67
CD	71	115	39	69	199	276	161	215	55
SCZ	109	349	36	54	33	113	26	35	45

*The number of unique genes mapped by one of the positional, eQTL and chromatin interaction mappings

Thus, using BMI summary statistics, FUMA confirmed known genes but also prioritized novel genes, including potential causal genes located outside the GWAS risk loci of BMI, which were missed in the original study.

### Application to Crohn’s disease GWAS

To further illustrate its utility, we applied FUMA to the summary statistics of Crohn’s disease^47^ (CD; 6333 cases and 15,056 controls). With FUMA, 95 lead SNPs from 184 independent significant SNPs across 71 genomic loci were identified for CD (Supplementary Fig. 3 and Supplementary Data 7–10). First, describing the results of positional mapping of deleterious coding SNPs and eQTL mapping, FUMA prioritized 95 unique genes from 32 loci (Supplementary Fig. 4), of which 39 genes were implicated by deleterious coding SNPs and 69 were implicated by eQTLs influencing expression of these genes (12 genes had both deleterious coding SNPs and eQTLs; Table 2 and Supplementary Data 11). The prioritized 95 genes include 37 known candidate genes that were also reported in the original study^47^ including well-known CD-related genes such as *NOD2*, *IL23R*, and *SLC22A5*, while 58 genes were novel (Supplementary Fig. 3; see Supplementary Note 4 and Supplementary Figs. 5–7 for detail results). These novel candidates include 18 genes that are physically located outside the GWAS risk loci, and the novel candidates mainly share immune system related biological functions with 37 previously known genes (Supplementary Data 12).

Chromatin interaction mapping using Hi-C data in small bowel and liver prioritized 199 genes of which 18 genes are overlapping with genes prioritized by positional and/or eQTL mappings and 149 genes are located outside of the genomic risk loci (Supplementary Data 11). That resulted in a total of 276 prioritized genes including 215 novel candidates which were not reported in the original study (Table 2 and Supplementary Fig. 3). Of the 23 loci which are mapped to at least one gene by positional and eQTL mappings, additional 23 loci are mapped to candidate genes by chromatin interaction mapping, in which several of the genes prioritized from those loci are involved in immune system and cytokine signaling pathways (Supplementary Fig. 4 and Supplemental Data 12). One of these 23 risk loci, the 17q12 locus is mapped to six chemokine ligands by Hi-C in liver: *CCL1*, *CCL2*, *CCL7*, *CCL8*, *CCL11*, and *CCL13*. Additionally, prioritized genes include 11 cytokines (*IL4*, *IL5*, *IL10*, *IL19*, *IL23R*, *IL24*, *IL27*, *IL33*, *IL1RL1*, *IL18R1*, and *IL18RAP*) wherein *IL18R1* and *IL18RAP* are also mapped by eQTLs in whole blood and *IL23R* and *IL27* are also mapped by deleterious coding SNPs which further supports the involvement of these cytokine genes in CD. The role of these chemokines and cytokines in inflammatory disease has been widely studied^48^ and yet, chromatin interaction mapping identified additional relevant candidates from the risk loci. The prioritized 276 genes showed enrichment in 123 canonical pathways such as immune system and cytokine related pathways which are known to be highly relevant to CD^49^ (Supplementary Data 13).

### Application to schizophrenia GWAS

We also applied FUMA to the most recent Schizophrenia (SCZ; 36,989 cases and 113,075 controls) GWAS summary statistics^3^, and 128 lead SNPs from 269 independent significant SNPs across 109 genomic loci were identified (Supplementary Note 5, Supplementary Fig. 8 and Supplementary Data 14–17). Positional mapping of deleterious coding SNPs and eQTL mapping prioritized 84 unique genes of which 36 genes were implicated by deleterious coding SNPs and 65 were implicated by eQTLs influencing expression of these genes (six genes had both deleterious coding SNPs and eQTLs; Supplementary Data 18). The prioritized 84 genes include 65 genes which were previously reported as candidates in the original study^3^, while 19 genes were novel (Table 2) including 11 genes which are physically located outside the GWAS risk loci. These 19 novel candidates have several shared biological functions with 65 previously known genes, such as “matrisome” and “neuronal system” (Supplementary Data 19). Out of 84 prioritized genes, 60 of them were also identified by the recent TWAS^50^ and Hi-C^51^ studies including 10 genes which are physically located outside the risk loci. The prioritized genes cover 34 genomic loci out of 109 of which 20 loci are mapped to single prioritized gene (Supplementary Fig. 9; see Supplementary Note 5 and Supplementary Fig. 10 for detailed results). These 20 genes are highly likely to drive the association signal in the genomic loci. These genes include *CACNA1C*, *LRP1*, *PLCB2*, *GRIN2A*, and *NMUR2*, which are involved in pathways such as Alzheimer’s disease, long-term potentiation, calcium signaling, and transmission across chemical synapses.

Chromatin interaction mapping using Hi-C data in hippocampus and prefrontal cortex prioritized 33 genes of which *DPYD* and *WBPIL* are also mapped by a deleterious coding SNP, and *VPS45* and *PITPNM2* are also mapped by eQTL in the brain (Supplementary Data 18). Out of these 33 genes, 15 are located outside of the genomic risk loci. Together with positional and eQTL mapping, this resulted in a total of 113 candidate genes including 35 novel candidates which are not reported in the original study (Table 2 and Supplementary Fig. 8). The 29 genes prioritized only by chromatin interactions have shared functions with other genes such as “regulation of response to stress” (*RWDD3*), “intracellular signal transaction” (*SGSM3*), and several functions involved in regulation of transcriptions (*OTUD7B* and *ZBTB18*; Supplementary Data 19).

Enrichment was seen in several brain-system related pathways, such as nicotinic acetylcholine receptors (nAChR), long-term potentiation and neurotransmitter receptor binding (Supplementary Data 20). nAChR is an important neuron receptor in which one of the subunits alpha-7 (*CHRNA7*) has been recently studied as a new Schizophrenia drug target^52,53^. nAChR was also identified as enriched pathway in the recent study using Hi-C in human cerebral cortex^51^ that suggests potential involvement of nAChR pathway in SCZ risk.

## Discussion

We introduce a web application named FUMA that allows to process GWAS summary statistics, and annotate, prioritize SNPs and genes and facilitates interpretation by providing interactive visualizations. FUMA provides a single platform that is built on the most popular tools for post-GWAS annotation and includes a rich collection of data repositories to bring insights into the phenotype of interest, and annotation in FUMA typically takes only ±30 min. For every prioritized gene, FUMA provides the rationale for pinpointing this gene, such as for example when the expression of the prioritized gene is altered by a SNP that is associated with the disease of interest. Interactive regional plots (Fig. 3 and Supplementary Figs. 5–7, 10) show which genes in a genomic risk locus are prioritized and which genes are not, and the annotated SNPs in the prioritized genes facilitate the generation of hypotheses for functional validation experiments. For example, if a gene is prioritized because of an associated loss-of-function SNP, follow-up validation experiments focusing on a knock-out of this gene may provide disease relevant functional information. On the other hand, if a gene is prioritized because a risk associated allele of a SNP increases expression of this gene in brain, then an overexpression experiment of this gene in neuronal cell cultures would be a more relevant experiment.

The availability of biological resources that can aid in the interpretation of GWAS results, such as Hi-C and ChIA-PET, have dramatically increased recently and several studies have identified novel candidates from GWAS risk loci by integrating their results for example with chromatin interactions^51,54–57^. These technologies have the potential to identify distal interactions of promoters and enhancers. Especially for risk loci for which it has been difficult to identify target genes due to the presence of gene desserts, distal interactions might point to causal gene. Indeed, we identified additional putative causal genes by performing chromatin interaction mapping on outcomes from three GWAS studies (BMI, CD, and SCZ) and the additionally identified genes based on chromatin interaction information were mostly located outside of the risk loci, and were shown to have shared function with known candidates. Although chromatin interactions are highly tissue/cell type specific, as well as time dependent, and currently available data is still limited in those aspects, FUMA provides an option to upload custom interaction matrices. Additionally, FUMA is built in such a way that newly published data including 3D chromatin interactions, eQTLs and other variant annotations can easily be included in the SNP2GENE process. This makes FUMA a flexible web tool which can be utilized not only for new GWAS results but also for previously published GWAS to re-annotate risk loci with the latest biological data sources.

In summary, FUMA provides an easy-to-use tool to functionally annotate, visualize, and interpret results from genetic association studies and to quickly gain insight into the directional biological implications of significant genetic associations. FUMA combines information of state-of-the-art biological data sources in a single platform to facilitate the generation of hypotheses for functional follow-up analysis aimed at proving causal relations between genetic variants and diseases.

## Methods

### Data pre-processing

All genetic data sets used in this study are based on the hg19 human assembly and rsIDs were mapped to dbSNP build 146 if necessary. To compute minor allele frequencies and LD structure, we used the data from the 1000 Genomes Project^27^ phase 3 (1000G). Minor allele frequency and *r* ^2^ of pairwise SNPs (minimum *r* ^2^ = 0.05 and maximum distance between a pair of SNPs is 1 Mb) were pre-computed using PLINK^26^ for each of available populations (AFR, AMR, EAS, EUR, and SAS). Functional annotations of SNPs were obtained from the following three repositories; CADD^13^, RegulomeDB^14^, and core 15-state model of chromatin^9,10,15^. Cis-eQTL information was obtained from the following four different data repositories; GTEx portal v6^8^, Blood eQTL browser^16^, BIOS QTL Browser^17^, and BRAINEAC^18^, and genes were mapped to ensemble gene ID if necessary (Supplementary Note 2). Pre-processed Hi-C data for 14 tissue types and seven cell lines were obtained from GSE87112^11^ (Supplementary Note 3). Predicted enhancer and promoter regions for 111 epigenomes were obtained from the Roadmap Epigenomics Projects^10^. Genomic coordinate of GWAS catalog^1^ reported SNPs was lifted down using liftOver software from hg38 to hg19. Normalized gene expression data (RPKM, Read Per Kilobase per Million) from GTEx portal v6^8^ for 53 tissue types were processed for different purposes. The details are described in “GTEx Gene Expression Data Set” section. Curated pathways and gene sets from MsigDB v5.2^21^ and WikiPathways^22^ which are assigned entrez ID.

### Characterization of genomic risk loci based on GWAS

To define genomic loci of interest to the trait based on provided GWAS summary statistics, pre-calculated LD structure based on 1000G of the relevant reference population (EUR for BMI, CD and SCZ) is used. First of all, independent significant SNPs with a genome-wide significant *P*-value (< 5e-8) and independent from each other at *r* ^2^ < 0.6 are identified. For each independent significant SNP, all known (i.e., regardless of being available in the GWAS input) SNPs that have *r* ^2^ ≥ 0.6 with one of the independent significant SNPs are included for further annotation (candidate SNPs). These SNPs may thus include SNPs that were not available in the GWAS input, but are available in the 1000G reference panel and are in LD with an independent significant SNP. Candidate SNPs can be filtered based on a user-defined minor allele frequency (MAF, ≥0.01 by default).

Based on the identified independent significant SNPs, independent lead SNPs are defined if they are independent from each other at *r* ^2^ < 0.1. Additionally, if LD blocks of independent significant SNPs are closely located to each other (< 250 kb based on the most right and left SNPs from each LD block), they are merged into one genomic locus. Each genomic locus can thus contain multiple independent significant SNPs and lead SNPs.

Besides using FUMA to determine lead SNPs based on GWAS summary statistics, users can provide a list of pre-defined lead SNPs. In addition, users can provide a list of pre-defined genomic regions to limit all annotations carried out by FUMA to those regions.

### Annotation of candidate SNPs in genomic risk loci

Functional consequences of SNPs on genes are obtained by performing ANNOVAR^12^ (“gene-based annotation”) using Ensembl genes (build 85). Note that SNPs can be annotated to more than one gene in case of intergenic SNPs which are annotated to the two closest up- and down-stream genes. CADD scores, RegulomeDB scores and 15-core chromatin state are annotated to all SNPs in 1000G phase 3 by matching chromosome, position, reference, and alternative alleles. eQTLs are also extracted by matching chromosome, position and alleles of all independent significant SNPs and SNPs which are in LD with one of the independent significant SNPs for each user-selected tissue type, wherein SNPs can have multiple eQTLs for distinct genes and tissue types (Supplementary Note 2). Information on previously known SNP-trait associations reported in the GWAS catalog is also retrieved for all SNPs of interest by matching chromosome and position.

### Gene mapping

Gene annotation is based on Ensembl genes (build 85). To match external gene IDs, ENSG ID is mapped to entrez ID yielding 35,808 genes which consist of 19,436 protein-coding genes, 9249 non-coding RNA, and other 7123 genes (e.g., pseudogenes, processed transcripts, immunoglobulin genes, and T-cell receptor genes).

Positional mapping is performed based on annotations obtained from ANNOVAR^12^. Two optional filters are provided to control the maximum distance from SNPs to genes and select specific functional consequences of SNPs on gene. When the former option is defined, FUMA maps SNPs to genes based on ANNOVAR annotation and a user-defined maximum distance is applied for intergenic SNPs. When the latter option is provided, FUMA maps only SNPs which have selected annotations annotated by ANNOVAR (e.g., coding or splicing SNPs).

For eQTL mapping, all independent significant SNPs and SNPs in LD of them are mapped to eQTLs in user-defined tissue types. By default, only significant SNP–gene pairs (false discovery rate (FDR)≤ 0.05) are used. Optionally, eQTLs can be filtered based on a user-defined *P*-value. eQTL mapping maps SNPs to genes up to 1 Mb apart (*cis*-eQTLs).

Chromatin interaction mapping is performed by overlapping independent significant SNPs and SNPs in LD of them with one end of significantly interacting regions in user-selected tissue/cell types. These SNPs are then mapped to genes whose promoter regions (250 bp up- and 500 bp down-stream of transcription start site by default) overlap with another end of the significant interactions. Optionally SNPs can be filtered for those overlapping with predicted enhancer regions of the user-selected epigenomes. Similarly, mapped genes can also be filtered for having promoter regions overlap with predicted promoter regions of the user-selected epigenomes.

Optional filtering of SNPs based on functional annotations obtained in step 2 of SNP2GENE (i.e., CADD score, RegulomeDB score, 15-core chromatin state) can be performed for positional, eQTL and chromatin interaction mappings separately. When any of these filters is activated, candidate SNPs are filtered primary to gene mapping. Note that this filtering of SNPs based on functional annotations for a certain mapping does not affect other mappings, e.g., when SNPs are filtered by CADD score in positional mapping but not in eQTL mapping, SNPs are filtered prior to positional mapping but eQTL mapping uses the original set of candidate SNPs.

For mapped genes, two scores of intolerance to functional mutations are annotated; probability of being loss-of-function intolerant (pLI)^58^ and non-coding residual variation intolerance score (ncRVIS)^59^.

### MAGMA for gene analysis and gene set analysis

FUMA uses input GWAS summary statistics to compute gene-based *P*-values (gene analysis) and gene set *P*-value (gene set analysis) using the MAGMA^35^ tool. For gene analysis, the gene-based *P*-value is computed for protein-coding genes by mapping SNPs to genes if SNPs are located within the genes. For gene set analysis, the gene set *P*-value is computed using the gene-based *P*-value for 4728 curated gene sets (including canonical pathways) and 6166 GO terms obtained from MsigDB v5.2. For both analyses, the default MAGMA setting (SNP-wise model for gene analysis and competitive model for gene set analysis) are used, and the Bonferroni correction (gene) or FDR (gene-set) was used to correct for multiple testing. 1000G phase 3^27^ is used as a reference panel to calculate LD across SNPs and genes.

### GTEx gene expression data set

Normalized gene expressions (reads per kilo base per million, RPKM) of 53 tissue types were obtained from GTEx (Supplementary Table 3). A total of 56,320 genes was available in GTEx, which we filtered on an average RPKM per tissue greater than or equal to 1 in at least one tissue type. This resulted in transcripts of 28,520 genes, of which 22,146 were mapped to entrez ID (see “Gene Mapping” section for details). In the GENE2FUNC, the heatmap of prioritized genes displays two expression values; (i) the average log2(RPKM+1) per tissue per gene, in which RPKM is winsorized at 50, allowing comparison of expression level across genes and tissue types and (ii) the average of the normalized expression (zero mean of log2(RPKM+1)) per tissue per gene allowing comparison of expression level across tissue types within a gene.

To obtain differentially expressed gene sets (DEG; genes which are significantly more or less expressed in a given tissue compared to others) for each of 53 tissue type, the normalized expression (zero mean of log2(RPKM+1)) is used. Two-sided Student’s *t*-tests are performed per gene per tissue against all other tissues. After the Bonferroni correction, genes with corrected *P*-value < 0.05 and absolute log fold change ≥ 0.58 are defined as a DEG set in a given tissue, i.e., for these gene expression in the given tissue had the largest discrepancy with expression in all other tissues. In addition, we distinguish between genes that are upregulated and downregulated in a specific tissue compared to other tissues, by taking the sign of *t*-score into account. In GENE2FUNC, genes are tested against those DEG sets by hypergeometric tests to evaluate if the prioritized genes (or a list of genes of interest) are overrepresented in DEG sets in specific tissue types.

### Gene set enrichment test

To test for overrepresentation of biological functions, the prioritized genes (or a list of genes of interest) are tested against gene sets obtained from MsigDB (i.e., hallmark gene sets, positional gene sets, curated gene sets, motif gene sets, computational gene sets, GO gene sets, oncogenic signatures, and immunologic signatures) and WikiPathways, using hypergeometric tests. The set of background genes (i.e., the genes against which the set of prioritized genes are tested against) is 19,283 protein-coding genes. Background genes can also be selected from gene types as described in the “Gene Mapping” section. Custom sets of background genes can also be provided by the users. Multiple testing correction (i.e., Benjamini–Hochberg by default) is performed per data source of tested gene sets (e.g., canonical pathways, GO biological processes, hallmark genes). FUMA reports gene sets with adjusted *P*-value ≤ 0.05 and the number of genes that overlap with the gene set > 1 by default.

### FUMA parameters for application to GWAS summary statistics

In the described applications, three mapping strategies were applied to GWAS summary statistics with the following settings: positional mapping was performed by selecting exonic and splicing SNPs with CADD score ≥ 12.37 (defined by Kircher et al.^13^) to restrict the mapping to deleterious coding SNPs. eQTL mapping was performed using GTEx eQTLs with FDR<0.05. Chromatin interaction mapping was performed using Hi-C data from Schmitt et al.^11^ and interactions were filtered by FDR<1e-6. Tissue types used for eQTLs and chromatin interaction mappings are described in the following section for each of three phenotypes. Other parameters not mentioned here were kept as default (Supplementary Table 2).

### Application to BMI GWAS

Parameters were set as described in the above section and we used eQTLs in 44 tissue types from GTEx. For chromatin interaction mapping, Hi-C data of 14 tissue types (Adrenal, Aorta, Bladder, Dorsolateral Prefrontal Cortex, Hippocampus, Left Ventricle, Liver, Lung, Ovary, Pancreas, Psoas, Right Ventricle, Small Bowel and Spleen) from GSE87112 was used. Indels were excluded. rsID was mapped to dbSNP build 146 and chromosome and positions were extracted based on human genome hg19 reference. Only protein-coding genes were used in gene mapping and enrichment of DEG in 53 tissue types, Canonical Pathways and GO terms were tested.

### Application to CD GWAS

We set parameters as described above and we used eQTLs in five tissue types from GTEx which are relevant to CD, i.e., Small Intestine, Colon Sigmoid, Colon Transverse, Stomach, and Whole Blood. Chromatin interaction mapping was performed using Hi-C data of two tissue types; Liver and Small Bowel from GSE87112. The MHC region and indels were excluded from the analysis. Since the input GWAS summary statistics only contained results from the discovery phase, we manually submitted the 71 reported lead SNPs to FUMA in addition to the independent lead SNPs that were identified as described above (Supplementary Data 7). Only protein-coding genes were used in mappings and enrichment of DEG in 53 tissue types, Canonical Pathways and GO terms were tested.

### Application to SCZ GWAS

Parameters were set as described above and eQTLs in 10 brain tissues from GTEx. Chromatin interaction mapping was performed using Hi-C data of two brain regions; hippocampus and prefrontal cortex. The extended MHC region (25–34 Mb), Chromosome X and indels were excluded from this analysis. The input GWAS summary statistics are based on the discovery phase and not all reported lead SNPs from the combined results of discovery and replication phases reached genome-wide significance. To include all reported lead SNPs, 111 non-indel lead SNPs were provided to FUMA and additional independent lead SNPs were identified at *P* < 5e-8 (Supplementary Data 14). Only protein-coding genes were used in mappings and enrichment of DEG in 53 tissue types, Canonical Pathways and GO terms were tested.

### Code availability

Source code of FUMA web application is available through a git repository at https://github.com/Kyoko-wtnb/FUMA-webapp/.

### Data availability

Data and tools used in FUMA are all publicly available from the following links (details are in Supplementary Table 1). dbSNP build 146 rsID archive: ftp.ncbi.nlm.nih.gov/snp/organisms/human_9606_b146_grch137p13/database/organism_data/RsMergeArch.bcp.gz, 1000 genome phase 3 reference panel: ftp.1000genomes.ebi.ac.uk/vol1/ftp/release/20130502/, CADD: http://cadd.gs.washington.edu/download, RegulomeDB: http://www.regulomedb.org/downloads, 15-core chromatin state: http://egg2.wustl.edu/roadmap/data/byFileType/chromhmmSegmentations/ChmmModels/coreMarks/jointModel/final/, GWAS catalog: https://www.ebi.ac.uk/gwas/, GTEx v6: http://www.gtexportal.org/home/, Blood eQTL Browser: http://genenetwork.nl/bloodeqtlbrowser/, BIOS QTL Browser: http://genenetwork.nl/biosqtlbrowser/, BRAINEAC: http://www.braineac.org/, HiC (GSE87112): https://www.ncbi.nlm.nih.gov/geo/query/acc.cgi?acc=GSE87112, promoter/enhancer regions: http://egg2.wustl.edu/roadmap/data/byDataType/dnase/, pLI score: ftp.broadinstitute.org/pub/ExAC_release/release0.3.1/functional_gene_constraint, ncRVIS score: http://journals.plos.org/plosgenetics/article/file?type=supplementary&id=info:doi/10.1371/journal.pgen.1005492.s011, MsigDB: http://software.broadinstitute.org/gsea/msigdb/, WikiPathways: http://wikipathways.org/index.php/WikiPathways, ANNOVAR: http://annovar.openbioinformatics.org/en/latest/, and MAGMA: https://ctg.cncr.nl/software/magma. GWAS summary statistics used in this study is available from the followings; BMI: http://portals.broadinstitute.org/collaboration/giant/index.php/GIANT_consortium_data_files, CD: ftp.sanger.ac.uk/pub/consortia/ibdgenetics/, SCZ: http://www.med.unc.edu/pgc/results-and-downloads.

## Electronic supplementary material

Supplementary Information

Peer review file

Description of additional supplementary files

Supplementary Data 1

Supplementary Data 2

Supplementary Data 3

Supplementary Data 4

Supplementary Data 5

Supplementary Data 6

Supplementary Data 7

Supplementary Data 8

Supplementary Data 9

Supplementary Data 10

Supplementary Data 11

Supplementary Data 12

Supplementary Data 13

Supplementary Data 14

Supplementary Data 15

Supplementary Data 16

Supplementary Data 17

Supplementary Data 18

Supplementary Data 19

Supplementary Data 20
