# Supplementary material for: Functional mapping and annotation of genetic associations with FUMA
Source: Nat Commun. 2017 Nov 28;8:1826. doi: 10.1038/s41467-017-01261-5 (PMC5705698; doi:10.1038/s41467-017-01261-5)
Supplement: Supplementary file 1 — Supplementary Information [file 41467_2017_1261_MOESM1_ESM.docx]

**Supplementary Note 1 Input and output format of FUMA**

As input, FUMA takes GWAS summary statistics as a plain text file. Since there are multiple applications which are widely used to perform GWAS such as PLINK, SNPTEST and METAL, to minimize input data formatting, FUMA automatically captures headers of the output files from these three tools. Users can also provide custom header names instead. Tab and single or multiple white spaces are accepted as delimiters.

The downloadable output files from both *SNP2GENE* and *GENE2FUNC* processes are tab delimited plain text files. The descriptions of each file are below.

**GenomicRiskLoci.txt**

Genomic risk loci defined by independent lead SNPs and maximum distance between their LD block.

- Genomic locus : Index of genomic rick loci.
- uniqID : Unique ID of SNPs consists of chr:position:allele1:allele2 where alleles are alphabetically ordered.
- rsID : rsID of the top lead SNP based on dbSNP build 146.
- chr : chromosome of top lead SNP
- pos : position of top lead SNP on hg19
- p : P-value of top lead SNP (from the input file).
- start : Start position of the locus
- end : End position of the locus
- nSNPs : The number of unique SNPs in the genomic locus, including non-GWAS-tagged SNPs (which are extracted from 1000G).
- nGWASSNPs : The number of the GWAS-tagged SNPs within the genomic locus. This is a subset of “nSNP”.
- nIndSigSNPs : The number of the independent significant SNPs in the genomic locus (r^2^ < user defined parameter)
- IndSigSNPs : rsID of independent significant SNPs in the genomic locus
- nLeadSNPs : The number of lead SNPs in the genomic locus (r^2^ < 0.1)
- LeadSNPs : rsID of lead SNPs in the genomic locus

**leadSNPs.txt**

lead SNPs defined by clumping independent SNPs at r^2^ 0.1.

- No : Index of lead SNPs
- Genomic Locus : Index of assigned genomic locus matched with "GenomicRiskLoci.txt". Multiple independent lead SNPs can be assigned to the same genomic locus.
- uniqID : Unique ID of SNPs consists of chr:position:allele1:allele2 where alleles are alphabetically ordered.
- rsID : rsID based on dbSNP build 146.
- chr : chromosome
- pos : position on hg19
- p : P-value (from the input file).
- nIndSigSNPs : The number of independent significant SNPs which are in LD of the lead SNP at r^2^ 0.1
- IndSigSNPs : rsID of independent significant SNPs which are in LD of the lead SNP at r^2^ 0.1

**IndSigSNPs.txt**

All independent significant SNPs at user defined r^2^.

- No : Index of independent significant SNPs
- Genomic Locus : Index of assigned genomic locus matched with "GenomicRiskLoci.txt". Multiple independent lead SNPs can be assigned to the same genomic locus.
- uniqID : Unique ID of SNPs consists of chr:position:allele1:allele2 where alleles are alphabetically ordered.
- rsID : rsID based on dbSNP build 146.
- chr : chromosome
- pos : position on hg19
- p : P-value (from the input file).
- nSNPs : The number of SNPs which are in LD of the independent significant SNP given r^2^, including non-GWAS-tagged SNPs (which are extracted from 1000G).
- nGWASSNPs : The number of GWAS-tagged SNPs which are in LD of the ind. sig. SNP given r^2^.

**snps.txt**

All SNPs in LD with any of independent lead SNPs with r^2^ greater or equal to the defined threshold.

- uniqID : Unique ID of SNPs consists of chr:position:allele1:allele2 where alleles are alphabetically ordered.
- rsID : rsID based on dbSNP build 146.
- chr : chromosome
- pos : position on hg19
- effect_allele : Effect allele if it is provided in the input GWAS summary statistics file. If not, this is the alternative (minor) allele in 1000G.
- non_effect_allele : Non-effect allele if it is provided in the input GWAS summary statistics file. If not, this is the reference allele in 1000G.
- MAF : Minor allele frequency computed based on 1000G.
- gwasP : P-value provided in the input GWAS summary statistics file. Non-GWAS tagged SNPs (which do not exist in input file but are extracted from the reference panel) have "NA" instead.
- or : Odds ratio provided in the input GWAS summary statistics file if available. Non-GWAS tagged SNPs (which do not exist in input file but are extracted from the reference panel) have "NA" instead.
- beta : Beta provided in the input GWAS summary statistics file if available. Non-GWAS tagged SNPs (which do not exist in input file but are extracted from the reference panel) have "NA" instead.
- se : Standard error provided in the input GWAS summary statistics file if available. Non-GWAS tagged SNPs (which do not exist in input file but are extracted from the reference panel) have "NA" instead.
- r2 : The maximum r^2^ of the SNP with one of the independent significant SNP (this doesn't have to be top lead SNPs in the genomic loci).
- IndSigSNP : rsID of a independent significant SNP which has the maximum r^2^ of the SNP.
- Genomic locus : Index of the genomic risk loci matching with "GenomicRiskLoci.txt".
- nearestGene : The nearest Gene of the SNP based on ANNOVAR annotations.
- Note that ANNOVAR annotates "consequence" function by prioritizing the most deleterious annotation for SNPs which are locating a genomic region where multiple genes are obverlapped.
- Genes are encoded in symbol, if it is available, otherwise Ensembl ID.
- Genes include all transcripts from Ensembl gene build 85 including non-protein coding genes and RNAs.
- dist : Distance to the nearest gene. SNPs which are locating in the gene body or 1kb up- or down-stream of TSS or TES have 0.
- func : Functional consequence of the SNP on the gene obtained from ANNOVAR. For exonic SNPs, detail annotation (e.g. non-synonymous, stop gain and so on) is available in ANNOVAR table (annov.txt).
- CADD : CADD score which is computed based on 63 annotations. The higher score, the more deleterious the SNP is. 12.37 is the suggested threshold by Kicher et al(ref).
- RDB : RegulomeDB score which is the categorical score (from 1a to 7). 1a is the highest score that the SNP has the most biological evidence to be regulatory element.
- minChrState : The minimum 15-core chromatin state across 127 tissue/cell type.
- commonChrState : The most common 15-core chromatin state across 127 tissue/cell types.
- posMapFilt : Whether the SNP was used for positional mapping or not. 1 is used, otherwise 0. When positional mapping is not performed, all SNPs have 0.
- eqtlMapFilt : Whether the SNP was used for eqtl mapping or not. 1 is used, otherwise 0. When eqtl mapping is not performed, all SNPs have 0.

**ld.txt**

r^2^ of all pairs of one of the independent lead SNPs and SNPs in LD with r^2^ greater or equal to the defined threshold.

- SNP1 : unique ID of one of the independent significant SNPs
- SNP2 : unique ID of SNPs which are in LD of SNP1 with r^2^ greater or equal to the user defined threshold
- r2 : r^2^ computed by plink using 1000 genomes reference panel (phase3) of defined population

**annov.txt**

Full results of ANNOVAR.

- uniqID : Unique ID of SNPs consists of chr:position:allele1:allele2 where alleles are alphabetically ordered.
- chr : chromosome
- pos : position on hg19
- Gene : Ensembl gene ID
- Symbol : Gene symbol
- Distance : Distance to the gene
- Function : Functional consequence on the gene
- Exonic function : Functional annotation of exonic SNPs
- Exon : Index of exon

**annot.txt**

annotations of all SNPs which are in LD of any independent lead SNP.

- uniqID : Unique ID of SNPs consists of chr:position:allele1:allele2 where alleles are alphabetically ordered.
- CADD : CADD score which is computed based on 67 annotations. The higher score, the more deleterious the SNP is. 12.37 is the suggested threshold by Kicher et al. (2014).
- RDB : RegulomeDB score which is the categorical score (from 1a to 7). 1a is the highest score that the SNP has the most biological evidence to be regulatory element.
- E001~E129 : Chromatin state predicted by ChrHMM.

**genes.txt**

The list of genes mapped by SNPs in snps.txt based on the user defined mapping parameters.

Columns with posMap, eqtlMap or ciMAp in the parentheses are only available when positional, eQTL or chromatin interaction mapping is performed, respectively.

- Gene : ENSG ID
- Symbol : Gene Symbol
- entrezID : entrez ID
- Genomic locus : Index of genomic loci where mapped SNPs are from. This could contain more than one interval in the case that eQTLs are mapped to genes from distinct genomic risk loci.
- chr : chromosome
- start : Starting position of the gene
- end : Ending position of the gene
- strand : Strand of the gene
- status : Status of the gene from Ensembl
- type : Gene biotype from Ensembl
- HUGO : HUGO (HGNC) gene symbol
- pLI : pLI score from ExAC database. The probability of being loss-of-function intolerant.
- posMapSNPs (posMap): The number of SNPs mapped to gene based on positional mapping (after functional filtering if parameters are given).
- posMapMaxCADD (posMap): The maximum CADD score of mapped SNPs by positional mapping.
- eqtlMapSNPs (eqtlMap): The number of SNPs mapped to the gene based on eQTL mapping.
- eqtlMapminP (eqtlMap): The minimum eQTL P-value of mapped SNPs.
- eqtlMapmin! (eqtlMap): The minimum eQTL FDR of mapped SNPs.
- eqtlMapts (eqtlMap): Tissue types of mapped eQTL SNPs.
- eqtlDirection (eqtlMap): consecutive direction of mapped eQTL SNPs.
- ciMap : If the gene is mapped by chromatin interaction "Yes", otherwise "No".
- minGwasP : The minimum P-value of mapped SNPs.
- IndSigSNPs : rsID of the all independent significant SNPs of mapped SNPs.

**eqtl.txt**

This file is only available when eQTL mapping is performed.

The file contains unique pair of SNP-gene-tissue, therefore, a SNP could appear multiple times.

- uniqID : Unique ID of SNPs consists of chr:position:allele1:allele2 where alleles are alphabetically ordered.
- chr : chromosome
- pos : position on hg19
- DB : Data source of eQTLs. Currently GTEx, BloodeQTL, BIOS and BRAINEAC are available.
- tissue : Tissue type
- Gene : ENSG ID
- Symbol : Gene symbol
- P-value : P-value of eQTLs
- FDR : FDR of eQTLs. Note that method to compute FDR differs between data sources.
- tz : T-statistics or z score depends on data source.

**ci.txt**

This file is only available when chromatin interaction mapping is performed.

- GenomicLocus : Index of genomic loci matching with “GenomicRiskLoci.txt”
- region1 : One end of a significant chromatin interaction which overlaps with at least one SNPs in one of the genomic risk loci.
- region2 : The other end of a significant chromatin interaction. This region could be located outside the risk loci.
- FDR : FDR of interaction.
- type : Type of chromatin interaction data, e.g. Hi-C or ChIA-PET
- DB : The name of the data source.
- tissue/cell : Tissue or cell type of the interaction.
- intra/inter : Intra- or Inter-chromosomal interaction.
- SNPs : rsID of SNPs overlapping with region 1.
- genes : ENSG ID of genes whose promoter regions overlap with region 2.

**ciSNPs.txt**

This file is only available when chromatin interaction mapping is performed.

The file contains SNPs in genomic risk loci that overlap with one end (region 1) of a significant chromatin interaction and enhancer regions of user selected tissue/cell types.

If no epigenome was selected, this file is empty.

- uniqID : Unique ID of SNPs consists of chr:position:allele1:allele2 where alleles are alphabetically ordered.
- rsID : rsID based on dbSNP build 146
- chr : chromosome
- pos : position on hg19
- reg_region : Predicted enhancer or dyadic regions
- type : enh for enhancer and dyadic for dyadic enhancer/promoter regions
- tissue/cell : EID of 111 Roadmap epigenomes

**ciProm.txt**

This file is only available when chromatin interaction mapping is performed.

The file contains promoter regions of user selected epigenomes (if selected) and genes whose promoter regions overlap.

- region2 : region 2 in "ci.txt" file
- reg_region : Predicted promoter or dyadic regions
- type : prom for promoter and dyadic for dyadic enhancer/promoter regions
- tissue/cell : EID of 111 Roadmap epigenomes
- genes : genes whose promoter regions overlap with region2

**gwascatalog.txt**

Information reported in GWAScatalog from SNPs in the GWAS risk loci.

- Genomic locus : Index of genomic risk loci.
- IndSigSNP : One of the independent significant SNPs of the SNP in GWAScatalog.
- chr : chromosome
- bp : position on hg19
- snp : rsID of reported SNP in GWAS catalog
- PMID : PubMed ID
- Trait : The trait reported in GWAScatalog
- FirthAuth : First author reported in GWAScatalog
- Date : Date added in GWAScatalog
- P-value : Reported P-value
- Journal : Abbreviated journal name
- Link : PubMed URL
- Study : Title of paper
- Trait : Disease or trait examined in study
- InitialN : Sample size and ancestry description for stage 1 of GWAS (summing across multiple Stage 1 populations, if applicable)
- ReplicationN : Sample size and ancestry description for subsequent replication(s) (summing across multiple populations, if applicable)
- Region : Cytogenetic region associated with rs number
- ReportedGene : Gene(s) reported by author
- MappendGene : Gene(s) mapped to the strongest SNP. If the SNP is located within a gene, that gene is listed. If the SNP is intergenic, the upstream and downstream genes are listed, separated by a hyphen.
- UpGene : Entrez Gene ID for nearest upstream gene to rs number, if not within gene
- DownGene : Entrez Gene ID for nearest downstream gene to rs number, if not within gene
- SNP_Gene_ID* : Entrez Gene ID, if rs number within gene; multiple genes denotes overlapping transcripts
- UpGeneDist : distance in kb for nearest upstream gene to rs number, if not within gene
- DownGeneDist : distance in kb for nearest downstream gene to rs number, if not within gene
- Strongest : SNP(s) most strongly associated with trait + risk allele (? for unknown risk allele). May also refer to a haplotype.
- SNPs : Strongest SNP; if a haplotype it may include more than one rs number (multiple SNPs comprising the haplotype)
- merged : denotes whether the SNP has been merged into a subsequent rs record (0 = no; 1 = yes;)
- SNP_ID_cur : current rs number (will differ from strongest SNP when merged = 1)
- Content : SNP functional class
- intergenic : denotes whether SNP is in intergenic region (0 = no; 1 = yes)
- RistAF : Reported risk/effect allele frequency associated with strongest SNP in controls (if not available among all controls, among the control group with the largest sample size). If the associated locus is a haplotype the haplotype frequency will be extracted.
- P : Reported p-value for strongest SNP risk allele (linked to dbGaP Association Browser). Note that p-values are rounded to 1 significant digit (for example, a published p-value of 4.8 x 10-7 is rounded to 5 x 10-7).
- Pmlog : -log(p-value)
- Ptext : Information describing context of p-value (e.g. females, smokers).
- OrBeta : Reported odds ratio or beta-coefficient associated with strongest SNP risk allele. Note that if an OR <1 is reported this is inverted, along with the reported allele, so that all ORs included in the Catalog are >1. Appropriate unit and increase/decrease are included for beta coefficients.
- 95CI : Reported 95% confidence interval associated with strongest SNP risk allele, along with unit in the case of beta-coefficients. If 95% CIs are not published, we estimate these using the standard error, where available.
- Platform : Genotyping platform manufacturer used in Stage 1; also includes notation of pooled DNA study design or imputation of SNPs, where applicable
- CNV : Study of copy number variation (yes/no)

**GS.txt**

Significantly enriched gene set tested in GENE2FUNC process.

- Category : Category of the gene set
- GeneSet : Name of the gene set
- N_genes : The number of genes in the gene set
- N_overlap : The number of input genes overlapped with the gene set
- p : hypergeometric statistics
- adjP : Adjusted P-value by user defined method
- genes : Symbols of input genes overlapped with the gene set
- link : Link to MsigDB

**DEG.txt / DEGgeneral.txt**

Tissue specificity of input genes tested in GENE2FUNC process by the hypergeometric.

- Category : Category of the gene set, DEG.up, DEG.down or DEG.twoside for up-regulated, down-regulated and both DEG gene set, respectively
- GeneSet : Tissue type
- N_genes : The number of genes in the gene set
- N_overlap : The number of input genes overlapped with the gene set
- p : hypergeometric statistics
- adjP : Adjusted P-value by user defined method
- genes : Ensembl gene ID of input genes overlapped with the gene set

**Supplementary Note 2 eQTL data sources**

eQTL datasets were obtained from four data repositories. From GTEx v6^1^, single tissue cis-eQTL data for every tested SNP-gene association for 44 tissue types was extracted (Supplementary Table 3). From Blood eQTL browser (5,311 peripheral blood samples)^2^, we obtained cis-eQTLs that were pre-filtered at FDR <0.50. From BIOS QTL browser (2,116 peripheral blood samples)^3^, gene-level cis-eQTLs were obtained and were pre-filtered at FDR <0.05. From BRAINEAC (134 individuals)^4^, cis-eQTLs of 10 brain regions were obtained and were pre-filtered at nominal P-value 0.05. Genes were mapped to ensembl gene ID. Tested alleles were not provided for BRAINEAC eQTLs (assigned NA).

Since FUMA contains 4 different eQTL data sources, there might be conflicting information, such that a SNPs is found to be an eQTL for a certain gene based on information from one repository but not the other. FUMA provides the evidence from the four different repositories as is, and users can decide to select only one repository or combine evidence from different repositories.

**Supplementary Note 3 3D chromatin interaction data**

Built-in Hi-C data of 14 tissue and 7 cell types were obtained from GSE87112^5^ in which the raw data was processed to intra chromosomal interactions at 40kb resolution. We used Fit-Hi-C output which computed the significance of interactions within binned genomic regions. As suggested by Schmitt et al., interactions are filtered at FDR <1e-6 by default. However, interactions significant at FDR <0.05 are also available in FUMA and can be obtained by modifying this parameter when submitting a job.

As an option, users can upload custom chromatin interaction matrices not limited to Hi-C but also 5C, ChIA-PET and Capture Hi-C. The input file for this is required to have the following 7 columns: (1) chromosome of region 1, (2) start position of region 1, (3) end position of region 1, (4) chromosome of region 2, (5) start position of region 2, (6) end position of region 2 and (7) parameter of significance of interaction such as FDR in which the order of region 1 and 2 are arbitral. Therefore, in the chromatin interaction mapping, the direction of interaction is not considered.

**Supplementary Note 4 Additional results for Crohn’s disease (CD)**

In one of the top hits of CD GWAS on 16q.12.1, FUMA confirmed association of a well-known CD gene, *NOD2*, by eQTLs in Whole Blood. We additionally identified *BRD7* which had eQTLs in Whole Blood, and *ADCY7* which had deleterious coding SNPs as candidate genes (Supplementary Fig. 5). *BRD7* and *ADCY7* were mapped by SNPs in the LD of distinct lead SNPs from the one which have SNPs in LD mapped to *NOD2*, suggesting multiple causal SNPs and affected genes in one genomic risk locus.

In the locus on 2p16.1 (chr2:61186829-61231014), *AHSA2* is identified as a single candidate gene which is about 200 Mb downstream of the top lead SNP (Supplementary Fig. 6). *AHSA2* was implicated by eQTLs in Colon Sigmoid, Colon Transverse and Whole Blood. Repnik and Potocnik recently reported that rs13003464 significantly alternates expression of *AHSA2* in Inflammatory Bowel Disease patients^6^. Despite of the relatively large number of genes closely located to the lead SNP, FUMA was able to pinpoint the most likely causal genes, by incorporating information on regulatory elements.

In the locus on 10q24.2 (chr10:101271789-101327851), FUMA identified one deleterious exonic SNP (rs41290504, CADD=15.6) of *NKX2-3* (Supplementary Fig.7). This SNP is not tagged in GWAS but identified from 1000G reference panel which is in strong LD with the top lead SNP (*r^2^*>=0.9 with rs4409764). Involvement of *NKX2-3* in the pathogenesis of CD has been suggested based on differential expression of this gene in CD patients compared to controls^7^.

**Supplementary Note 5 Additional results for Schizophrenia (SCZ) GWAS summary statistics**

The number of genomic risk loci identified in this study is slightly different from 108 loci reported in the original study^8^ because *i.* indels and X chromosome were removed in our analysis, *ii.* there are a few SNPs that achieved genome wide significance in the discovery phase but did not do so in combined statistics, and therefore were not reported in the original study*.* As the publicly available GWAS summary statistics is the discovery results, these SNPs are still included. *iii.* MHC region was excluded in our study due to long high-LD structure.

In the second-most significant association in SCZ GWAS on 10q.24.32, there are 19 protein-coding genes located within the genomic interval. FUMA prioritized 5 of these (Supplementary Fig. 10); 3 genes included a deleterious coding SNPs in LD with the lead SNP (*CYP17A1*, *CNNM2* and *WBP1L*) and two genes were implicated through eQTLs in brain in LD with the lead SNP (*NT5C2* and *ARL3*).

**Supplementary Table 1. Data repositories and tools used in FUMA**

| Category | Name | Description | Last accessed | Link |
| --- | --- | --- | --- | --- |
| Reference Variants | dbSNP 146 | Map rsID of input files to dbSNP build 146 | 18-Mar-16 | <ftp://ftp.ncbi.nlm.nih.gov/snp/organisms/human_9606_b146_grch137p13/database/organism_data/RsMergeArch.bcp.gz> |
| Reference Genome | 1000 Genomes Project Phase3 | Compute MAF and *r^2^* for each available population | 25-Apr-16 | <ftp://ftp.1000genomes.ebi.ac.uk/vol1/ftp/release/20130502/> |
| Functional annotations of SNPs | CADD v1.3 | Deleteriousness score of variants | 09-Jul-16 | <http://cadd.gs.washington.edu/download> |
|  | RegulomeDB | Score of regulatory variants | 16-Feb-16 | <http://www.regulomedb.org/downloads> |
|  | 15-core chromatin state | Chromatin states of genomic region in 127 tissue/cell types | 15-May-16 | <http://egg2.wustl.edu/roadmap/data/byFileType/chromhmmSegmentations/ChmmModels/coreMarks/jointModel/final/> |
|  | GWAS catalog | Known trait associated variants | 05-Oct-16 | <https://www.ebi.ac.uk/gwas/> |
| eQTLs | GTEx v6 | cis-eQTLs of 44 tissue types | 16-Mar-16 | <http://www.gtexportal.org/home/> |
|  | Blood eQTL Browser | cis-eQTLs of blood cell | 06-Jun-16 | <http://genenetwork.nl/bloodeqtlbrowser/> |
|  | BIOS QTL Browser | cis-eQTLs of blood cell | 07-Oct-16 | <http://genenetwork.nl/biosqtlbrowser/> |
|  | BRAINEAC | cis-eQTLs of 10 brain regions | 09-Sep-16 | <http://www.braineac.org/> |
| HiC | GSE87112 | HiC data for 14 tissue types and 7 cell lines | 24-Apt-17 | <https://www.ncbi.nlm.nih.gov/geo/query/acc.cgi?acc=GSE87112> |
| Regulatory elements | Roadmapc epigenomics project | Enhancer, promoter and dyadic enhancer/promoter regions in 111 epigenomes | 24-Apt-17 | <http://egg2.wustl.edu/roadmap/data/byDataType/dnase/> |
| Gene score | pLI | Probability of being loss-of-function intolerance | 24-Apt-17 | <ftp://ftp.broadinstitute.org/pub/ExAC_release/release0.3.1/functional_gene_constraint> |
|  | ncRVIS | Non-coding residual variation intolerance score | 24-Apt-17 | <http://journals.plos.org/plosgenetics/article/file?type=supplementary&id=info:doi/10.1371/journal.pgen.1005492.s011> |
| Gene expression | GTEx v6 | Normalized gene expression (RPKM: Read Per Kilo base per Million) for 53 tissue types | 16-Mar-16 | <http://www.gtexportal.org/home/> |
| Gene sets | MsigDB v5.2 | Curated pathways and gene sets | 26-Dec-16 | <http://software.broadinstitute.org/gsea/msigdb/> |
|  | WikiPathways | Curated pathways | 22-Apr-16 | <http://wikipathways.org/index.php/WikiPathways> |
| Tools | ANNOVAR | Variant annotation tool | 11-Feb-16 | <http://annovar.openbioinformatics.org/en/latest/> |
|  | MAGMA v6.0 | Software for gene-based test and gene-set analyses of GWAS | 17-Jan-17 | <https://ctg.cncr.nl/software/magma> |

**Supplementary Table 2. Parameters of FUMA (SNP2GENE)**

| Category | Mandatory/Optional | Parameter | Description | Default |
| --- | --- | --- | --- | --- |
| Identification of lead SNPs | Mandatory | leadP (<) | The maximum P-value to be lead SNPs | 5.00E-08 |
|  | Mandatory | r^2^ (>=) | The minimum threshold to be in LD | 0.6 |
|  | Mandatory | gwasP (<) | The maximum P-value to be selected as candidate SNPs | 0.05 |
|  | Mandatory | population | The population for reference panel | EUR |
|  | Optional | MAF (>=) | The minimum minor allele frequency | 0.01 |
|  | Optional | Include 1KG SNPs | If checked, all 1000G SNPs with *r^2^* greater than threshold will be included into candidate SNPs. | Yes |
|  | Optional | mergeDist (<) | The maximum distance between LD blocks to merge into a genomic locus. | 250 |
|  | Optional | Additional SNPs identification | When predefined lead SNPs are provided, whether identify additional lead SNPs based on leadP. | Yes |
| Gene mapping | Mandatory | positional mapping | Whether perform positional mapping | TRUE |
|  | Optional | distance based mapping | To map SNPs to genes based on physical distance | Yes |
|  | Mandatory if distance based mapping is active | window (<) | The maximum distance to map SNPs to genes | 10kb |
|  | Optional | annotation | SNPs positional annotation to map to genes | all |
|  | Mandatory | eQTL mapping | Whether perform eQTL mapping | FALSE |
|  | Mandatory if eQTL mapping is true | tissue | Tissue type to use for eQTL mapping | all |
|  | Mandatory if eQTL mapping is true | significant eQTL only | Whether map only significant eQTL at FDR < 0.05 | TRUE |
|  | Optional if significant eQTL only is FALSE | eqtlP (<) | The maximum (nominal) P-value of eQTL | 1.00E-03 |
|  | Mandatory | chromatin interaction mapping | Whether perform chromatin interaction mapping | FALSE |
|  | Optional | build in chromatin interaction data | Build in Hi-C data for chromatin interaction mapping | None |
|  | Mandatory if chromatin interaction mapping is true | FDR of interaction (<) | The maximum FDR of significance of chromatin interactions | 1.00E-06 |
|  | Mandatory if chromatin interaction mapping is true | Promoter regions | User defined promoter region around TSS of genes | 250bp up- and 500bp down-stream of TSS |
|  | Optional | Cell/tissue types of enhancer and promoter regions | Cell/tissue types of 111 epigenomes to annotate predicted enhancer and promoter regions to interacting regions | None |
|  | Optional | Use enhancers for filtering | Whether filter SNPs in one end of a significant interaction on which are overlapped with enhancer regions of selected epigenomes | FALSE |
|  | Optional | Use promoters for filtering | Whether filter genes whose promoter regions are overlapped with predicted promoter regions of selected epigenomes | FALSE |
|  | Optional* | CADD (>=) | The minimum CADD score | 0 |
|  | Optional* | RDB (<=) | The minimum RegulomeDB score | 7 |
|  | Optional* | Chromatin state filtering | Whether filter SNPs based on chromatin 15 states | FALSE |
|  | Optional* | Cell/tissue types | Cell/tissue type of chromatin state | none |
|  | Optional* | chromatin state (<=) | The minimum chromatin state | 7 |
|  | Optional* | Method for chromatin state filtering | When multiple tissue/cell types are selected, this method will apply to filter SNPs | none |

*Options are available for each of three mapping strategy separately.

**Supplementary Table 3. Tissue types available in FUMA**

| General Tisssue | Tissue | Sample size | Genotyped sample size |
| --- | --- | --- | --- |
| Adipose_Tissue | Adipose_Subcutaneous | 350 | 298 |
| Adipose_Tissue | Adipose_Visceral_Omentum | 227 | 185 |
| Adrenal_Gland | Adrenal_Gland | 145 | 126 |
| Bladder | Bladder | 11 | 11 |
| Blood | Cells_EBV-transformed_lymphocytes | 118 | 114 |
| Blood | Whole_Blood | 393 | 197 |
| Blood_Vessel | Artery_Aorta | 224 | 118 |
| Blood_Vessel | Artery_Coronary | 133 | 285 |
| Blood_Vessel | Artery_Tibial | 332 | 338 |
| Brain | Brain_Amygdala | 72 | 62 |
| Brain | Brain_Anterior_cingulate_cortex_BA24 | 84 | 72 |
| Brain | Brain_Caudate_basal_ganglia | 117 | 100 |
| Brain | Brain_Cerebellar_Hemisphere | 105 | 89 |
| Brain | Brain_Cerebellum | 125 | 103 |
| Brain | Brain_Cortex | 114 | 96 |
| Brain | Brain_Frontal_Cortex_BA9 | 108 | 92 |
| Brain | Brain_Hippocampus | 94 | 81 |
| Brain | Brain_Hypothalamus | 96 | 81 |
| Brain | Brain_Nucleus_accumbens_basal_ganglia | 113 | 93 |
| Brain | Brain_Putamen_basal_ganglia | 97 | 82 |
| Brain | Brain_Spinal_cord_cervical_c-1 | 71 | 59 |
| Brain | Brain_Substantia_nigra | 63 | 56 |
| Breast | Breast_Mammary_Tissue | 214 | 183 |
| Cervix_Uteri | Cervix_Ectocervix | 6 | 6 |
| Cervix_Uteri | Cervix_Endocervix | 5 | 5 |
| Colon | Colon_Sigmoid | 149 | 124 |
| Colon | Colon_Transverse | 196 | 169 |
| Esophagus | Esophagus_Gastroesophageal_Junction | 153 | 127 |
| Esophagus | Esophagus_Mucosa | 286 | 241 |
| Esophagus | Esophagus_Muscularis | 247 | 218 |
| Fallopian_Tube | Fallopian_Tube | 6 | 6 |
| Heart | Heart_Atrial_Appendage | 194 | 159 |
| Heart | Heart_Left_Ventricle | 218 | 190 |
| Kidney | Kidney_Cortex | 32 | 26 |
| Liver | Liver | 119 | 97 |
| Lung | Lung | 320 | 278 |
| Muscle | Muscle_Skeletal | 430 | 361 |
| Nerve | Nerve_Tibial | 304 | 256 |
| Ovary | Ovary | 97 | 85 |
| Pancreas | Pancreas | 171 | 149 |
| Pituitary | Pituitary | 103 | 87 |
| Prostate | Prostate | 106 | 87 |
| Salivary_Gland | Minor_Salivary_Gland | 57 | 51 |
| Skin | Cells_Transformed_fibroblasts | 284 | 272 |
| Skin | Skin_Not_Sun_Exposed_Suprapubic | 250 | 196 |
| Skin | Skin_Sun_Exposed_Lower_leg | 357 | 302 |
| Small_Intestine | Small_Intestine_Terminal_Ileum | 88 | 77 |
| Spleen | Spleen | 104 | 89 |
| Stomach | Stomach | 193 | 170 |
| Testis | Testis | 172 | 157 |
| Thyroid | Thyroid | 323 | 278 |
| Uterus | Uterus | 83 | 70 |
| Vagina | Vagina | 96 | 79 |

Tissues which are only available for gene expression data but not for eQTLs (genotyped sample size < 70) are shaded.

**
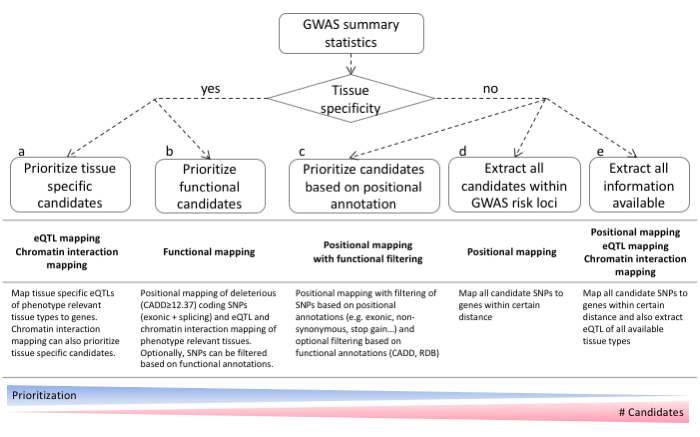
**

**Supplementary Figure 1. Guideline of Gene Mapping**

Schematic overview that depicts information retrieval based on prior knowledge. (a) Prioritization of tissue specific candidate genes by eQTLs and chromatin interactions in (expected) tissue types. (b) Prioritization of genes by combining all positional, eQTL and chromatin interaction mappings with filtering on functional SNPs. (c) Prioritization of genes by positional mapping with filtering on functional SNPs. (d) Extraction of all genes locating within a risk loci by Naïve positional mapping. (e) Full extracting of possible candidates by combining GWAS risk loci, and eQTLs across all available tissue types.

**
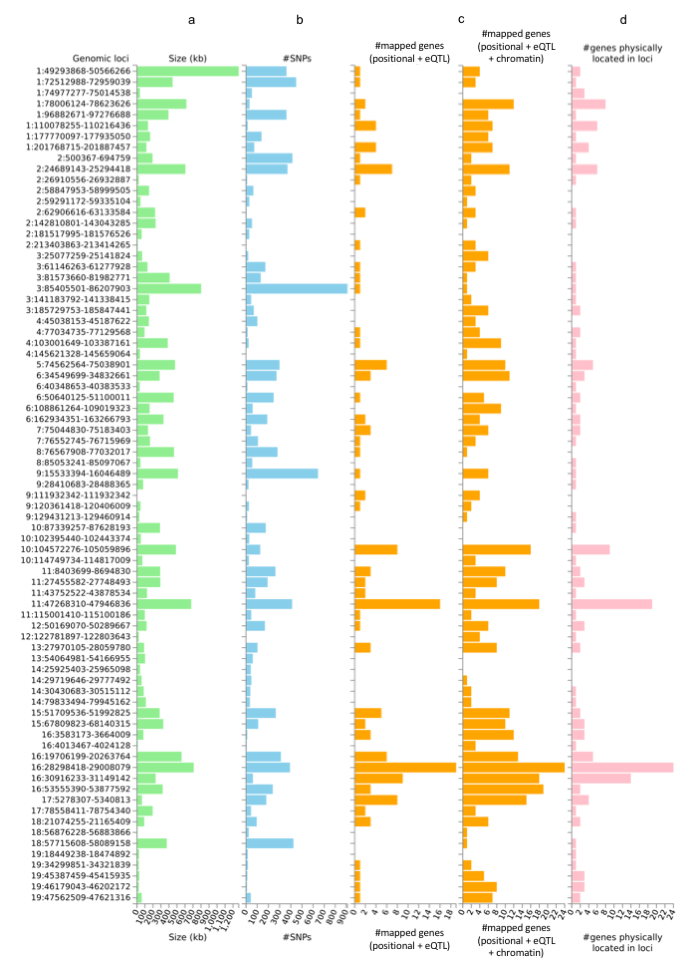
**

**Supplementary Figure 2. Summary of genomic risk loci of BMI GWAS**

Genomic risk loci are represented by ‘chromosome:start position-end position’ on Y axis. Histograms depict, (a) the size of the genomic locus, (b) the number of candidate SNPs in the genomic locus, (c) the number of mapped genes by the positional and eQTL mappings (left), and all three mappings including chromatin interaction mapping (right) in the genomic locus and (d) the number of genes known to be located within the genomic loci.

**
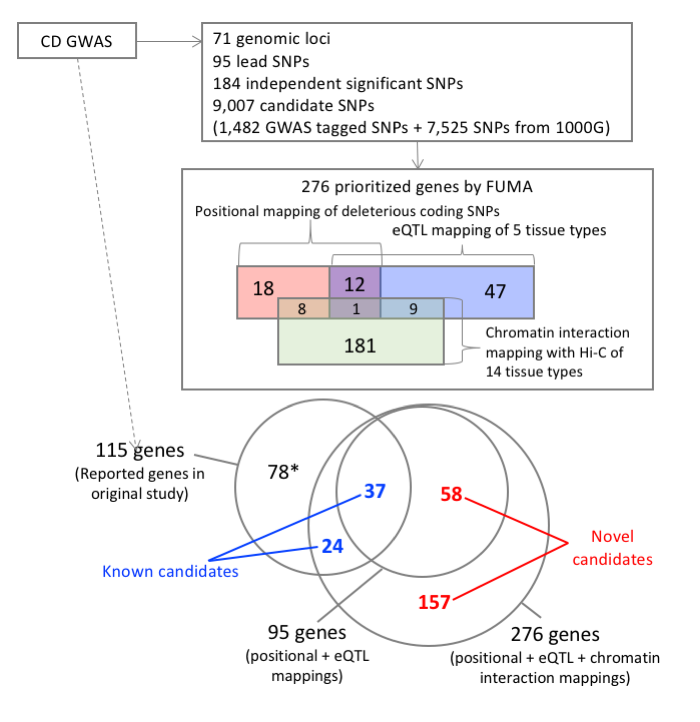
**

**Supplementary Figure 3. Summary of results for CD GWAS**

Starting from the CD GWAS summary statistics, boxes represent results of SNP2GENE process. Candidate SNPs include all independent lead SNPs and SNPs which are in LD with these lead SNPs. Prioritized genes are divided into three categories; genes that were implicated by candidate SNPs which are deleterious coding SNPs (colored pink), eQTLs for these genes (colored blue) or by chromatin interactions (colored green). Genes implicated by both strategies are colored purple. The prioritized genes are further categorized into previously reported genes (blue) and novel genes (red) prioritized genes by FUMA. ^*^These genes were not prioritized by FUMA since they do not have deleterious coding SNPs, eQTLs or chromatin interactions, although they are located within GWAS risk loci.

**
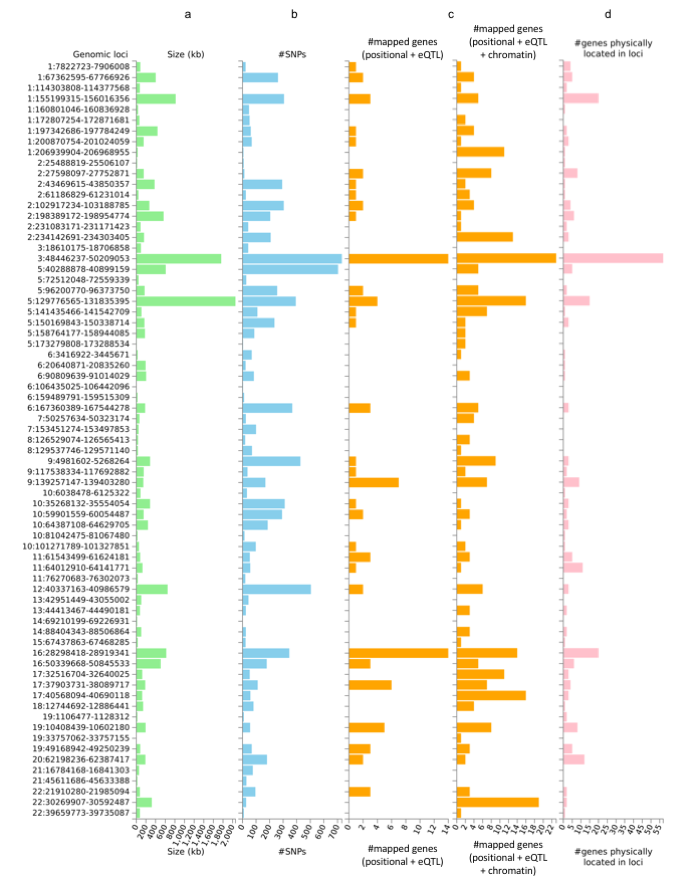
**

**Supplementary Figure 4. Summary of genomic risk loci of CD GWAS**

Genomic risk loci are represented by ‘chromosome:start position-end position’ on Y axis. Histograms depict, (a) the size of the genomic locus, (b) the number of candidate SNPs in the genomic locus, (c) the number of mapped genes by the positional and eQTL mappings (left), and all three mappings including chromatin interaction mapping (right) in the genomic locus and (d) the number of genes known to be located within the genomic loci.

**
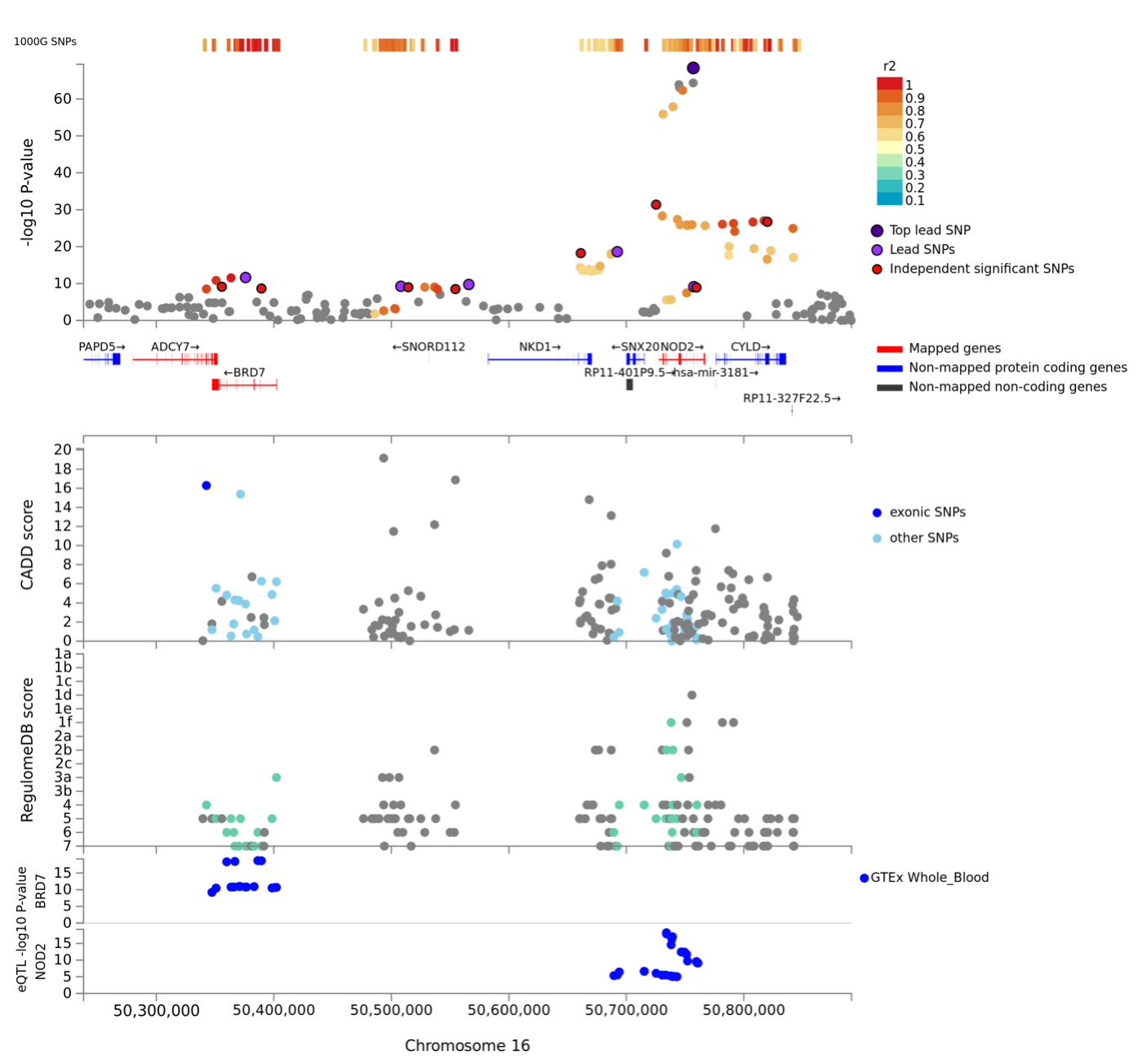
**

**Supplementary Figure 5. Regional plot of locus on 16q12.1 of CD GWAS**

Regional plot of NOD2 locus on 16q12.1. Genes prioritized by FUMA are highlighted in red. From the top, GWAS P-value (SNPs are colored based on *r^2^*), CADD score (coding SNPs and other SNPs are colored blue and light blue, respectively), RequlomeDB score and eQTL P-value. Non-GWAS-tagged SNPs are shown in the top of the plot as rectangles since they do not have a P-value from the GWAS, but they are in LD with the lead SNP. eQTLs are plotted per gene and colored based on tissue types. In the plots of CADD score, RegulomeDB score and eQTLs, SNPs which are not mapped to any gene are colored grey.

**
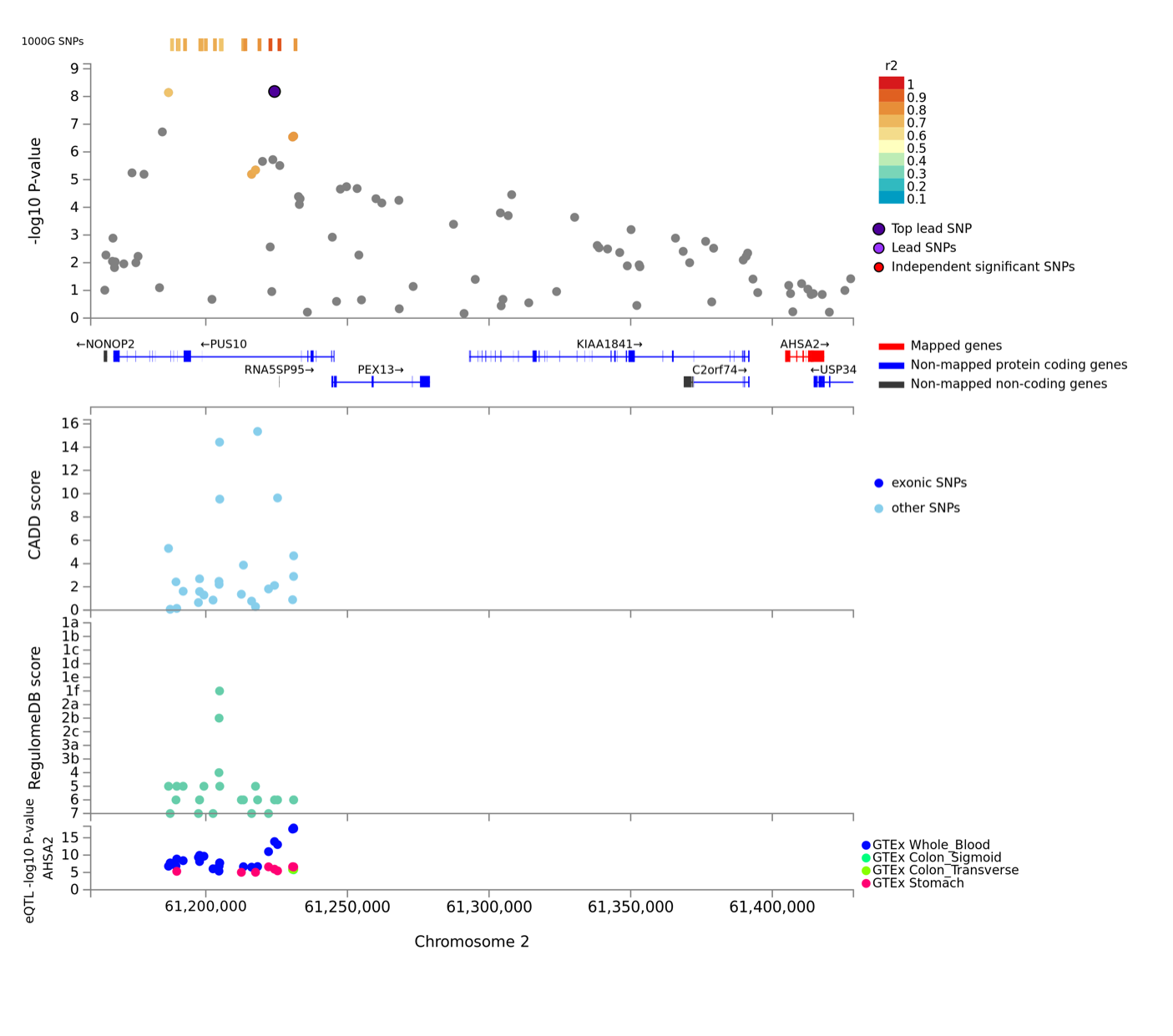
**

**Supplementary Figure 6. Regional plot of locus on 2p16.1 of CD GWAS**

Regional plot of locus on 2p.16.1. Genes prioritized by FUMA are highlighted in red. From the top, GWAS P-value (SNPs are colored based on *r^2^*), CADD score, RequlomeDB score and eQTL P-value. Non-GWAS-tagged SNPs are shown in the top of the plot as rectangles since they do not have a P-value from the GWAS, but they are in LD with the lead SNP. eQTLs are plotted per gene and colored based on tissue types. In the plots of CADD score, RegulomeDB score and eQTLs, SNPs which are not mapped to any gene are colored grey.

**
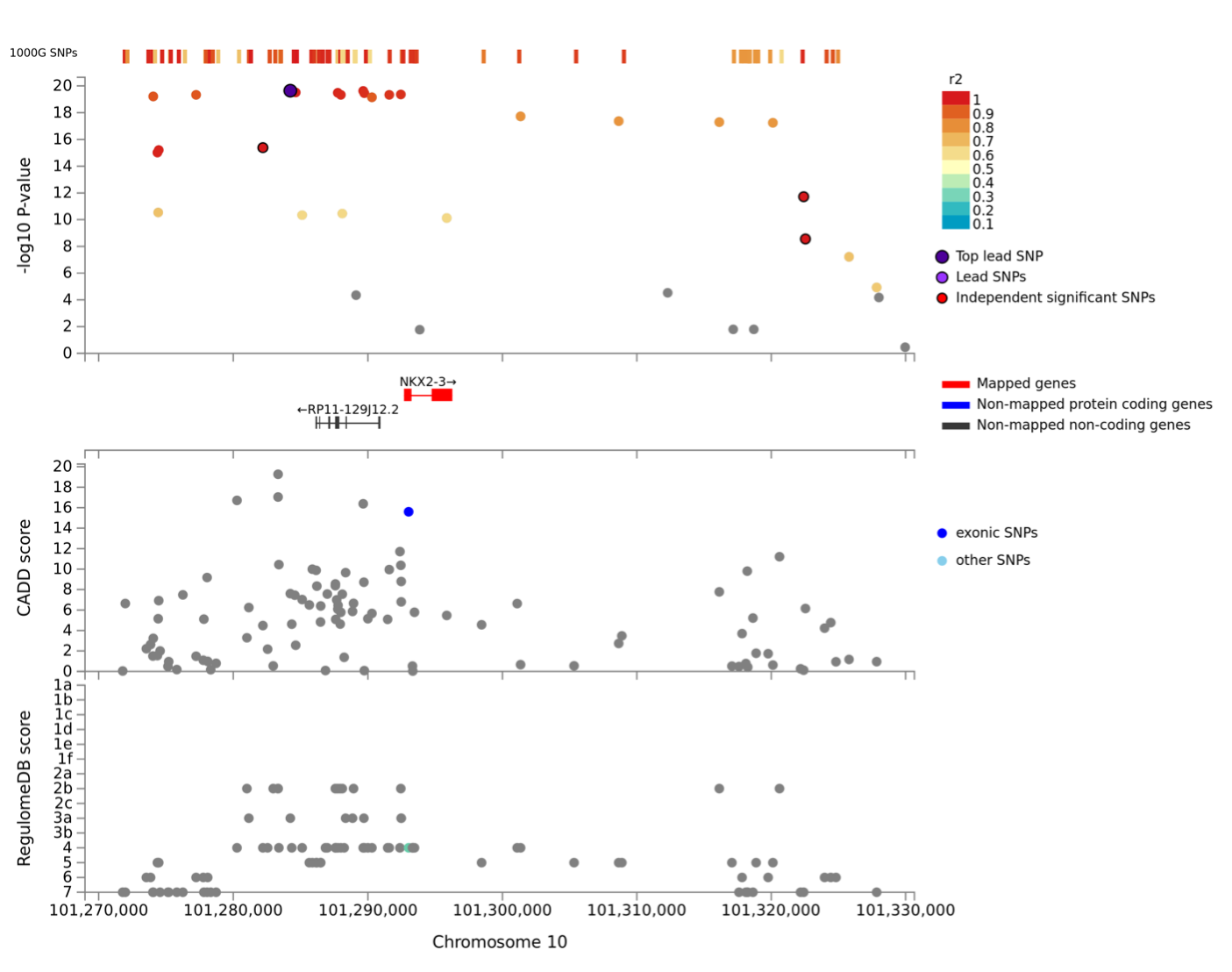
**

**Supplementary Figure 7. Regional plot of locus on 10q24.2 of CD GWAS**

Regional plot of locus 10q24.2. Genes prioritized by FUMA are highlighted in red. From the top, GWAS P-value (SNPs are colored based on *r^2^*), CADD score (coding SNPs and other SNPs are colored blue and light blue, respectively) and RequlomeDB score. Non-GWAS-tagged SNPs are shown in the top of the graph as rectangles since they do not have a P-value from the GWAS, but they are in LD with the lead SNP. In the plots of CADD score, RegulomeDB score and eQTLs, SNPs which are not mapped to any gene are colored grey.

**
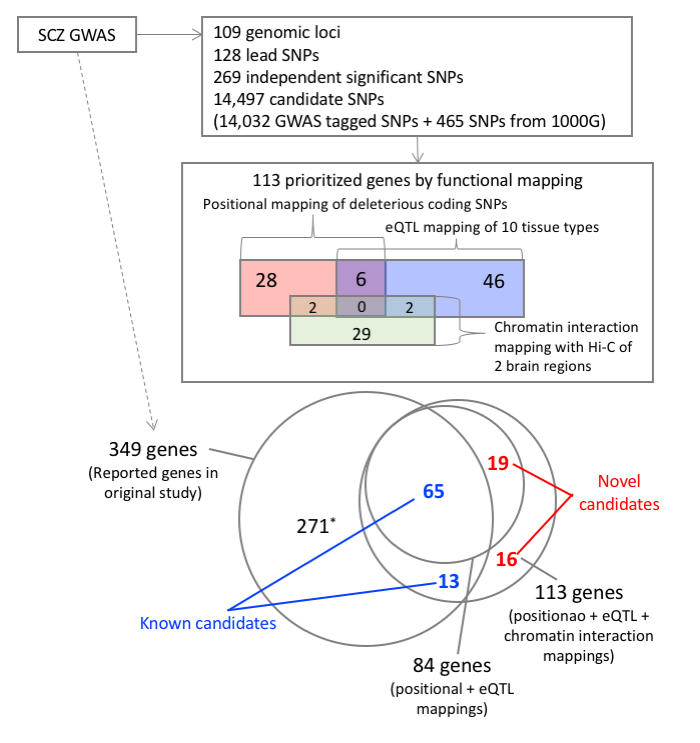
**

**Supplementary Figure 8.** **Summary results for SCZ GWAS**

Starting from the CD GWAS summary statistics, boxes represent results of SNP2GENE process. Candidate SNPs include all independent lead SNPs and SNPs which are in LD with these lead SNPs. Prioritized genes are divided into three categories; genes that were implicated by candidate SNPs which are deleterious coding SNPs (colored pink), eQTLs for these genes (colored blue) or chromatin interactions (colored green). Genes implicated by both strategies are colored purple. The prioritized genes are further categorized into previously reported genes (blue) and novel genes (red) prioritized genes by FUMA. *These genes were not prioritized by FUMA since they do not have either deleterious coding SNPs, eQTLs or chromatin interactions, although they are located within GWAS risk loci.

**
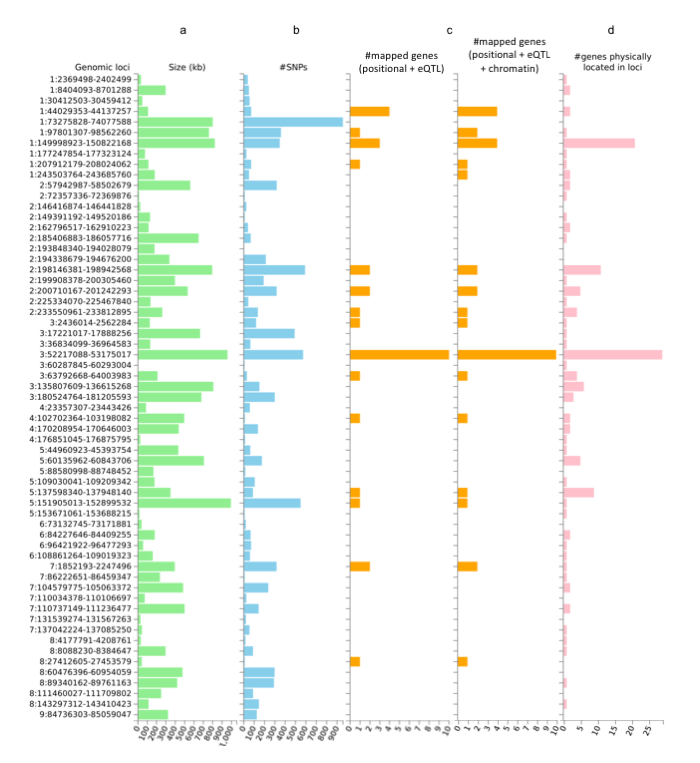
**

**
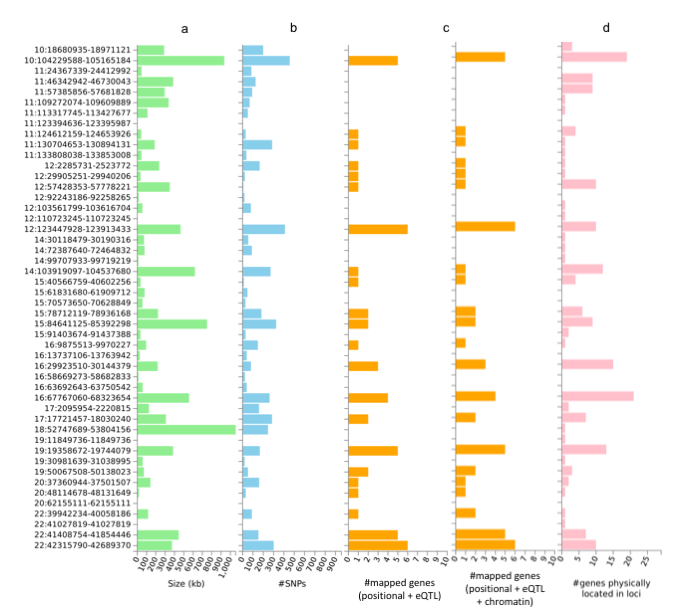
**

**Supplementary Figure 9. Summary of genomic risk loci of SCZ GWAS**

Genomic risk loci are represented by ‘chromosome:start position-end position’ on Y axis. Histograms depict, (a) the size of the genomic locus, (b) the number of candidate SNPs in the genomic locus, (c) the number of mapped genes by the positional and eQTL mappings (left), and all three mappings including chromatin interaction mapping (right) in the genomic locus and (d) the number of genes known to be located within the genomic loci.

**
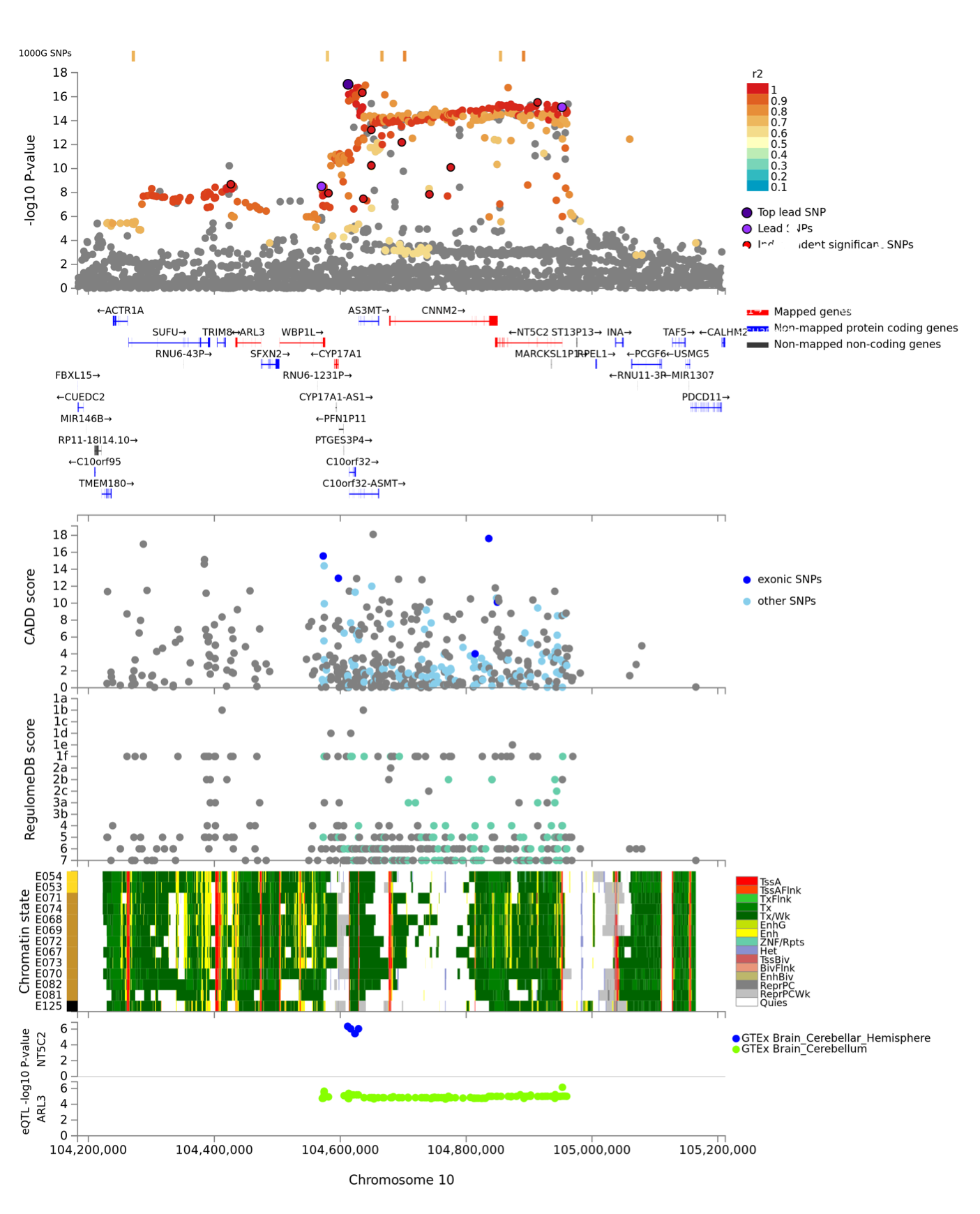
**

**Supplementary Figure 10. Regional plot of locus on 10q24.32 of SCZ GWAS**

Regional plot of locus 10q24.32. Genes prioritized by FUMA are highlighted in red. From the top, GWAS P-value (SNPs are colored based on *r^2^*), CADD score (coding SNPs and other SNPs are colored blue and light blue, respectively) and RequlomeDB score, 15-core chromatin state in the brain and eQTL P-value. Non-GWAS-tagged SNPs are shown in the top of the plot as rectangles since they do not have a P-value from the GWAS, but they are in LD with the lead SNP. Tissue/cell types of epigenome ID are the following; E054: Ganglion eminence derived primary cultured neurospheres, E053: Cortex derived primary cultured neurospheres, E071: Hippocampus middle, E074: Substantia nigra, E068: Anterior caudate, E069: Cingulate gyrus, E072: Inferior temporal lobe, E067: Angular gyrus, E073: Dorsolateral prefrontal cortex, E070: Germinal matrix, E082: Fetal brain female, E081: Fetal brain male and E125: NH-A Astrocytes primary cells. eQTLs are plotted per gene and colored based on tissue types. In the plots of CADD score, RegulomeDB score and eQTLs, SNPs which are not mapped to any gene are colored grey.

**References**

1. The GTEx Consortium. The Genotype-Tissue Expression (GTEx) pilot analysis: Multitissue gene regulation in humans. *Science (80-. ).* **348,** 648–660 (2015).

2. Westra, H.-J. *et al.* Systematic identification of trans eQTLs as putative drivers of known disease associations. *Nat. Genet.* **45,** 1238–43 (2013).

3. Zhernakova, D. V *et al.* Identification of context-dependent expression quantitative trait loci in whole blood. *Nat. Genet.* **49,** 139–145 (2016).

4. Ramasamy, A. *et al.* Genetic variability in the regulation of gene expression in ten regions of the human brain. *Nat. Neurosci.* **17,** 1418–28 (2014).

5. Schmitt, A. D. *et al.* A Compendium of chromatin contact maps reveals spatially active regions in the human genome. *Cell Rep* **17,** 2042–2059 (2016).

6. Repnik, K. & Potočnik, U. eQTL analysis links inflammatory bowel disease associated 1q21 locus to ECM1 gene. *J. Appl. Genet.* **57,** 363–372 (2016).

7. Yu, W. *et al.* Association of a Nkx2-3 polymorphism with Crohn’s disease and expression of Nkx2-3 is up-regulated in B cell lines and intestinal tissues with Crohn’s disease. *J. Crohn’s Colitis* **3,** 189–195 (2009).

8. Ripke, S. *et al.* Biological insights from 108 schizophrenia-associated genetic loci. *Nature* **511,** 421–427 (2014).
