# Supplementary material for: Functional mapping and annotation of genetic associations with FUMA
Source: Nat Commun. 2017 Nov 28;8:1826. doi: 10.1038/s41467-017-01261-5 (PMC5705698; doi:10.1038/s41467-017-01261-5)
Supplement: Supplementary file 3 — Description of additional supplementary files [file 41467_2017_1261_MOESM3_ESM.pdf]

Description of Additional Supplementary Files

File Name: Supplementary Data 1
Description: Independent significant SNPs at r2<0.6 identified from BMI GWAS.
Genomic locus: the index of genomic risk loci matched with S. Data 3, #SNPs in LD: The number of SNPs which are in LD of the corresponding independent significant SNPs at r2<0.6. This includes the SNPs that are not available in the input of GWAS summary statistics but are extracted from 1000G, #GWAS SNPs in LD: This is the subset of #SNPs in LD that are available in the input GWAS summary statistics. They are filtered on P-value <=0.05.

File Name: Supplementary Data 2
Description: Lead SNPs identified from independent significant SNPs of BMI GWAS.
Genomic locus: the index of genomic risk loci matched with S. Data 3, #Ind. Sig. SNPs: The number of independent significant SNPs which are in LD with the corresponding lead SNPs at r2>=0.1, Ind. Sig. SNPs: rsID of independent significant SNPs which are in LD of the corresponding lead SNPs at r2>=0.1.

File Name: Supplementary Data 3
Description: Genomic risk loci of interest from BMI GWAS. chr, bp, p: chromosome, position and GWAS P-value of the top lead SNP (SNP with the minimum P-value) in the genomic locus, #SNPs in LD: The total number of unique SNPs which are in LD with one of the independent lead SNPs merged into the corresponding genomic loci. This includes SNPs that are not available in the input of GWAS summary statistics but are extracted from 1000G, #GWAS SNPs in LD: This is the subset of #SNPs within LD which are avaialble in the input GWAS summary statistics. They are filtered on P-value ≤ 0.05, #Ind. Sig. SNPs: The number of independent significant SNPs in the genomic locus, Ind. Sig. SNPs: rsID of independent significant SNPs in the genomic locus, #Lead SNPs: The number of lead SNPs in the genomic locus, Lead SNPs: rsID of lead SNPs in the genomic locus.

File Name: Supplementary Data 4
Description: Prioritized genes from BMI GWAS by functional mapping.
Genes only mapped by chroamtin interaction mapping are highlighted in blue. Genomic locus: The index of genomic risk loci matched with S. Data 3, candidate: "KNOWN" if the gene is reported in the original GWAS study, "NOVEL" otherwise, type: gene biotype obtained from Ensembl BioMart, pLI: pLI score obtained from ExAC database. The probability of being loss-of-function intolerant, ncRVIS: Non-coding residual variation intolerance score. The higher the score is, the more intolerant to non-coding variants the gene is, posMapSNPs: The number of SNPs mapped to the gene by positional mapping, posMapMaxCADD: The maximum CADD score of SNPs mapped to the gene by positional mapping, eqtlMapSNPs: The number of unique SNPs mapped to the gene by eQTL mapping. Note that SNPs can have multiple eQTLs of the same gene in different tissue types but they are counted one time per gene, eqtlMapminP: The minimum eQTL P-value of mapped SNPs to the gene, eqtlMapminQ: The minimum eQTL Q-value (gene Q-value of GTEx eQTLs) of mapped SNPs to the gene, eqtlMapts: The tissue types of eQTLs mapped to the gene,

eqtlDirection: The consensus (majority) direction of eQTLs. '+' for up-regulation and '-' for down-regulation, ciMap: "Yes" if the gene is mapped by chroamatin interaction mapping, "No" otherwise, ciMapts: Tissue types of chromatin interactions, ciMapOnly: 1 if the gene is only mapped by chromatin interactions, 0 otherwise, minGWASp: The minimum GWAS P-value of mapped SNPs of the corresponding genes. 'NA' means there was no GWAS tagged SNPs in mapped SNPs, Ind. sig. SNPs: rsID of independent significant SNPs of all mapped SNPs, matched with S. Data 1.

File Name: Supplementary Data 5
Description: Shared biological functions between known and novel candidate genes prioritized by FUMA from BMI GWAS.
For GO terms, only terms with less than 500 genes in total are shown. Known candidates: The prioritized genes (without chromatin interaction mapping) which were reported in the original GWAS study, Novel candidates: The prioritized genes (without chromatin interaction mapping) which were not reported in the original GWAS study, Known candidates (ciMap): The additionally prioritized genes by chromatin interaction mapping which were reported in the original GWAS study, Novel candidates (ciMap): The additionally prioritized genes by chromatin interaction mapping which were not reported in the original GWAS study, N: The total number of prioritized genes (known + novel) in the gene set, Total: The total number of genes in the gene set.

File Name: Supplementary Data 6
Description: Enriched gene sets by prioritized genes (without chromatin interaction mapping) from BMI GWAS.
FDR: BH corrected P-value (per category).

File Name: Supplementary Data 7
Description: Pre-defined lead SNPs for CD GWAS.

File Name: Supplementary Data 8
Description: Independent significant SNPs at $r^2<0.6$ identified from CD GWAS.
Genomic locus: the index of genomic risk loci matched with S. Data 10, #SNPs in LD: The number of SNPs which are in LD of the corresponding independent significant SNPs at $r^2<0.6$. This includes the SNPs that are not available in the input of GWAS summary statistics but are extracted from 1000G, #GWAS SNPs in LD: This is the subset of #SNPs in LD that are available in the input GWAS summary statistics. They are filtered on P-value <=0.05.

File Name: Supplementary Data 9
Description: Lead SNPs identified from independent significant SNPs of CD GWAS.
Genomic locus: the index of genomic risk loci matched with S. Data 10, #Ind. Sig. SNPs: The number of independent significant SNPs which are in LD with the corresponding lead SNPs

at r2>=0.1, Ind. Sig. SNPs: rsID of independent significant SNPs which are in LD of the corresponding lead SNPs at r2>=0.1.

File Name: Supplementary Data 10
Description: Genomic risk loci of interest from CD GWAS.
chr, bp, p: chromosome, position and GWAS P-value of the top lead SNP (SNP with the minimum P-value) in the genomic locus, #SNPs in LD: The total number of unique SNPs which are in LD with one of the independent lead SNPs merged into the corresponding genomic loci. This includes SNPs that are not available in the input of GWAS summary statistics but are extracted from 1000G, #GWAS SNPs in LD: This is the subset of #SNPs within LD which are avaialble in the input GWAS summary statistics. They are filtered on P-value ≤ 0.05, #Ind. Sig. SNPs: The number of independent significant SNPs in the genomic locus, Ind. Sig. SNPs: rsID of independent significant SNPs in the genomic locus, #Lead SNPs: The number of lead SNPs in the genomic locus, Lead SNPs: rsID of lead SNPs in the genomic locus.

File Name: Supplementary Data 11
Description: Prioritized genes from CD GWAS by functional mapping.
Genes only mapped by chromatin interaction mapping are highlighted in blue. Genomic locus: The index of genomic risk loci matched with S. Data 3, candidate: "KNOWN" if the gene is reported in the original GWAS study, "NOVEL" otherwise, type: gene biotype obtained from Ensembl BioMart, pLI: pLI score obtained from ExAC database. The probability of being loss-of-function intolerant, ncRVIS: Non-coding residual variation intolerance score. The higher the score is, the more intolerant to non-coding variants the gene is, posMapSNPs: The number of SNPs mapped to the gene by positional mapping, posMapMaxCADD: The maximum CADD score of SNPs mapped to the gene by positional mapping, eqtlMapSNPs: The number of unique SNPs mapped to the gene by eQTL mapping. Note that SNPs can have multiple eQTLs of the same gene in different tissue types but they are counted one time per gene, eqtlMapminP: The minimum eQTL P-value of mapped SNPs to the gene, eqtlMapminQ: The minimum eQTL Q-value (gene Q-value of GTEx eQTLs) of mapped SNPs to the gene, eqtlMapts: The tissue types of eQTLs mapped to the gene, eqtlDirection: The consensus (majority) direction of eQTLs. '+' for up-regulation and '-' for down-regulation, ciMap: "Yes" if the gene is mapped by chroamatin interaction mapping, "No" otherwise, ciMapts: Tissue types of chromatin interactions, ciMapOnly: 1 if the gene is only mapped by chromatin interactions, 0 otherwise, minGWASp: The minimum GWAS P-value of mapped SNPs of the corresponding genes. 'NA' means there was no GWAS tagged SNPs in mapped SNPs, Ind. sig. SNPs: rsID of independent significant SNPs of all mapped SNPs, matched with S. Data 8.

File Name: Supplementary Data 12
Description: Shared biological functions between known and novel candidate genes prioritized by FUMA from CD GWAS.
For GO terms, only terms with less than 500 genes in total are shown. Known candidates: The prioritized genes (without chromatin interaction mapping) which were reported in the original GWAS study, Novel candidates: The prioritized genes (without chromatin interaction mapping) which were not reported in the original GWAS study, Known candidates (ciMap):

The additionally prioritized genes by chromatin interaction mapping which were reported in the original GWAS study, Novel candidates (ciMap): The additionally prioritized genes by chromatin interaction mapping which were not reported in the original GWAS study, N: The total number of prioritized genes (known + novel) in the gene set, Total: The total number of genes in the gene set.

File Name: Supplementary Data 13
Description: Enriched gene sets by prioritized genes (without chromatin interaction mapping) from CD GWAS.
FDR: BH corrected P-value (per category).

File Name: Supplementary Data 14
Description: Pre-defined lead SNPs for SCZ GWAS.

File Name: Supplementary Data 15
Description: Independent significant SNPs at r2<0.6 identified from SCZ GWAS. Genomic locus: the index of genomic risk loci matched with S. Data 17, #SNPs in LD: The number of SNPs which are in LD of the corresponding independent significant SNPs at r2<0.6. This includes the SNPs that are not available in the input of GWAS summary statistics but are extracted from 1000G, #GWAS SNPs in LD: This is the subset of #SNPs in LD that are available in the input GWAS summary statistics. They are filtered on P-value <=0.05.

File Name: Supplementary Data 16
Description: Lead SNPs identified from independent significant SNPs of SCZ GWAS. Genomic locus: the index of genomic risk loci matched with S. Data 17, #Ind. Sig. SNPs: The number of independent significant SNPs which are in LD with the corresponding lead SNPs at r2>=0.1, Ind. Sig. SNPs: rsID of independent significant SNPs which are in LD with the corresponding lead SNPs at r2>=0.1.

File Name: Supplementary Data 17
Description: Genomic risk loci of interest from SCZ GWAS.
chr, bp, p: chromosome, position and GWAS P-value of the top lead SNP (SNP with the minimum P-value) in the genomic locus, #SNPs in LD: The total number of unique SNPs which are in LD with one of the independent lead SNPs merged into the corresponding genomic loci. This includes SNPs that are not available in the input of GWAS summary statistics but are extracted from 1000G, #GWAS SNPs in LD: This is the subset of #SNPs within LD which are avaialble in the input GWAS summary statistics. They are filtered on P-value ≤ 0.05, #Ind. Sig. SNPs: The number of independent significant SNPs in the genomic locus, Ind. Sig. SNPs: rsID of independent significant SNPs in the genomic locus, #Lead SNPs: The number of lead SNPs in the genomic locus, Lead SNPs: rsID of lead SNPs in the genomic locus.

File Name: Supplementary Data 18
Description: Prioritized genes from SCZ GWAS by functional mapping.
Genes only mapped by chromatin interaction mapping are highlighted in blue. Genomic locus: The index of genomic risk loci matched with S. Data 3, candidate: "KNOWN" if the gene is reported in the original GWAS study, "NOVEL" otherwise, type: gene biotype obtained from Ensembl BioMart, pLI: pLI score obtained from ExAC database. The probability of being loss-of-function intolerant, ncRVIS: Non-coding residual variation intolerance score. The higher the score is, the more intolerant to non-coding variants the gene is, posMapSNPs: The number of SNPs mapped to the gene by positional mapping, posMapMaxCADD: The maximum CADD score of SNPs mapped to the gene by positional mapping, eqtlMapSNPs: The number of unique SNPs mapped to the gene by eQTL mapping. Note that SNPs can have multiple eQTLs of the same gene in different tissue types but they are counted one time per gene, eqtlMapminP: The minimum eQTL P-value of mapped SNPs to the gene, eqtlMapminQ: The minimum eQTL Q-value (gene Q-value of GTEx eQTLs) of mapped SNPs to the gene, eqtlMapts: The tissue types of eQTLs mapped to the gene, eqtlDirection: The consensus (majority) direction of eQTLs. '+' for up-regulation and '-' for down-regulation, ciMap: "Yes" if the gene is mapped by chroamatin interaction mapping, "No" otherwise, ciMapts: Tissue types of chromatin interactions, ciMapOnly: 1 if the gene is only mapped by chromatin interactions, 0 otherwise, minGWASp: The minimum GWAS P-value of mapped SNPs of the corresponding genes. 'NA' means there was no GWAS tagged SNPs in mapped SNPs, Ind. sig. SNPs: rsID of independent significant SNPs of all mapped SNPs, matched with S. Data 15.

File Name: Supplementary Data 19
Description: Shared biological functions between known and novel candidate genes prioritized by FUMA from SCZ GWAS.
For GO terms, only terms with less than 500 genes in total are shown. Known candidates: The prioritized genes (without chromatin interaction mapping) which were reported in the original GWAS study, Novel candidates: The prioritized genes (without chromatin interaction mapping) which were not reported in the original GWAS study, Known candidates (ciMap): The additionally prioritized genes by chromatin interaction mapping which were reported in the original GWAS study, Novel candidates (ciMap): The additionally prioritized genes by chromatin interaction mapping which were not reported in the original GWAS study, N: The total number of prioritized genes (known + novel) in the gene set, Total: The total number of genes in the gene set.

File Name: Supplementary Data 20
Description: Enriched gene sets by prioritized genes (without chromatin interaction mapping) from SCZ GWAS.
FDR: BH corrected P-value (per category).
