# Supplementary material for: Functional mapping and annotation of genetic associations with FUMA
Source: Nat Commun. 2017 Nov 28;8:1826. doi: 10.1038/s41467-017-01261-5 (PMC5705698; doi:10.1038/s41467-017-01261-5)
Supplement: Supplementary file 2 — Peer review file [file 41467_2017_1261_MOESM2_ESM.pdf]

Reviewer #1 (Remarks to the Author):

#2017.4.2 FUMA: Functional mapping and annotation of genetic associations

The manuscript described a web-based toolset, FUMA, to annotate significant locus of genome-wide association studies (GWAS). FUMA incorporated several databases and bioinformatics softwares to develop a pipeline that help users to better analyze GWAS results and pick up likely functional locus, both in coding regions and regulatory regions; while, in the mean time, provide easy visualization tools.

The tool is needed in the field; The databases chosen for the genomic annotation is comprehensive though straightforward; The design of the website is clear and functional. However, the novelty of the work is a concern, since all the bioinformatics tools and databases used by FUMA are well-established and the annotation process of FUMA is standard. FUMA glues the resources together and provides the whole analysis pipeline as a service.

There is room for the improvement of the website. The regional plots that align the annotation of multiple databases, such as CADD, regulomeDB, and Chromatine states, is informative. However, it is desired that FUMA could provide clickable links that direct the users to more detailed information at the original databases. For example, regulomeDB also provide information on cell types and transcription factor names, instead of merely a score. In my test run, there are glitches on some pages where texts and figures stacked on top of each other, making it impossible to read.

Reviewer #2 (Remarks to the Author):

Most genetic variants associated with human disease sit in non-coding regions, but the function of these regions is hard to annotate and understand, a factor that challenges modern genetic studies of disease. It is in this context that the authors create an important new tool, FUMA for functional mapping and annotation of genetic variants associated with disease. They use this in a very striking proof of principle to identify new loci for BMI using published GWAS. Overall, I expect that this will be a widely referenced and highly cited paper, both for the new biology and for the tool. Most of my suggestions for improvement should therefore be considered minor, since this is an exciting advance that will substantially augment current genetic analyses.

The tool has two components, SNP to gene and gene to function, which are the key steps in moving from genetic association to some functional insight. I discuss each of these steps within this software

and its application to BMI in turn. The SNP to gene mapping divides LEAD SNPs into those that are clearly functional within a gene, and those that have no clear function, which are than annotated based on eQTL data. FUMA can calculate LEAD SNPs based itself based on certain (reasonable) assumptions, or take a list generated by the User, which is a key feature, since other methods such as CAVIAR or PAINTOR etc. may be used to define potentially causal SNP lists, and one needs flexibility in such a tool, since methods evolve so rapidly. This makes this a very flexible tool for its SNP to gene function, which will promote its use.

Moving from gene to function is a more far more involved and difficult problem. ANNOVAR is used to annotate SNPS, as well as CADD, Regulome DB and HMM based chromatin state from ENCODE. Non-coding or otherwise non-annotated SNPs are than matched with eQTLs from user defined tissues of interest, which is very useful. It is not clear if there are multiple eQTL data sets available, how they are merged, or reconciled if they give different or conflicting information. This can be easily addressed in the supplemental material.

One weakness of the mapping SNP to gene using ANNOVAR is that it is based on distance, and both functional eQTL and physical, Hi-C data indicate that many even intragenic SNPs act at distal genes. Assignment of non-coding SNPs to the closest gene is estimated to occur about 50% of the time, so 50% of the assignments might be off. The authors should consider integrating Hi-C/ChIA-PET and other data that is available via 3D genome browser (http://promoter.bx.psu.edu/hi-c/) or one of the other visualization tools, which would bring this pipeline to be truly state of the art. There should at least be a way to load a 2D matrix of chromatin contacts defined by Hi-C or refined by CTCF binding sites combined with other computational predictions, etc (e.g. PMID: 27064255). Since Supplemental figure 1 has a box that says “extract all information possible”, I suggest that the authors provide a means to incorporate or extract enhancer – promotor interactions from either Hi-C data, or computational predictions (PMID: 27064255), ATAC-seq correlations, or all of the above. Again, this would mean adding a SNP to gene annotation step that could accommodate a 2D interaction matrix, adding flexibility similar to the ability to load in a SNP list in the beginning of the process.

Similarly, CADD has not proven that powerful for identifying missense mutations in several disorders, even though it was originally published in Nature Genetics (the current FUMA software is likely to be much more useful!). Annotation with other tools that incorporate mutational frequency and locus tolerance to damage (which seems more promising) would be useful (PMID: 26332131; PMID: 25086666), but since this field is rapidly evolving, making sure that other emerging forms of functional annotation as to the likely deleteriousness of the mutation, both non-coding and coding, can be added would be very important. If the software is available as open source, that would also aid such additions and building on to this tool in the future.

Finally, with regards to the tool's functionality, the integration and use of MAGMA, which is a very strong and widely used tool for pathway annotation is strength of this current work.

The authors subsequently use this tool to re-annotate BMI GWAS, which adds substantial new biology and demonstrates the power of this tool by identifying 96 new putative candidate genes missed in the original study and 22 loci implicating single genes, several of which are new. While individually these individual genes need additional evidence, as a group, they fall into the same functional categories as the original GWAS identified, providing confidence that this FUMA is adding substantial value. Further, the demonstration of an eQTL locus overlapping a particular risk locus, provides a demonstration of the functionality of the locus, as long as the same SNP is implicated. In this regard, it would be useful to know whether direct overlap between the two is needed (same SNP or surrogate in both), or whether it is locus overlap, which is slightly less convincing.

The authors also apply FUMA to SCZ and IBD/CD GWAS, which is presented in the supplementary material. This shows similar results to the IBD in that new loci are identified, and they are plausible.

With regards to these applications, at least a summary in the main manuscript rather than only supplemental – such as the number of new loci and pathways in each (in a figure or table etc) would enhance the manuscript. A key question with regards to the SCZ analysis is how it is supported by recent Hi-C data (PMID:27760116and TWAS (doi: https://doi.org/10.1101/067355). Both the TWAS and Hi-C identify many genes outside the LD block that defines these SCZ loci.

The authors also identify ACH signaling as a pathway, which is interesting, and was also identified in the Hi-C work – that could be emphasized and cited as it is an external validation of this current work, as long as the loci actually overlap.

Reviewer #3 (Remarks to the Author):

The web service (FUMA) presented by Watanabe et al combines a number of typical steps frequently taken in the analysis of GWAS data. They have illustrated their framework at the example of three representative data set (BMI, CD, SCZ) to produce priority lists and new suggestions for "causal" genes.

Novelty.

The work includes suggestions for new genes that may be of interest for specialists working on the respective diseases. But none of the new genes were validated or evaluated beyond a cursory literature screen. Also for the computational aspects, I could not identify any novel methodological developments or components. Popular tools and data resources have been combined. While I recognise the importance of such efforts, it is a major omission that prior works in the field of GWAS analysis have not been cited. There is even an old review (Hou & Zhao, Front Genet. 2013) that includes many tools and web services for GWAS prioritisation. A thorough comparison with such works is necessary to assess the usefulness of this new web server.

Accessibility.

In general it would be preferable to use their service without registration, and to offer login (and perhaps an analysis history) only as an option for recurring users. When I had tried to login as example4@fuma.nl I got an error (see attached file). Therefore I could not evaluate the web service other than through the descriptions in the manuscript.

As is common practice for larger collection of data and annotations, it would also be useful to have an API for more programmatic access and batch processing.

Sustainability.

Undoubtedly extensive efforts went into collecting and preparing the various data sources for functional annotation of SNPs. Therefore it would be important to understand if, and to what extent, this process has been automated. Otherwise the service will soon be outdated and unmaintainable by the onslaught of new data and changing annotations. It would be equally important to describe in more detail the infrastructure and computational resources which are in place to sustain this service long-term.

Data formats

Throughout the manuscript the data formats were unclear or imprecise. I assume that the input "GWAS summary statistics" refers to VCF files, but which version? Similarly, also the output formats and standards should be defined more carefully.

Below we provide a point-by-point response indicating the changes made to the manuscript. For clarity, our response is written in blue.

Editor's comments

Q1.

We are particularly interested in the possibility of incorporating chromatin conformation data into the tool, as suggested by Reviewer #2.

A1.

We have now incorporated chromatin interaction mapping in the tool (see our detailed answer to Q9 and Q10).

Q2.

The other concerns mentioned by R1 and R3 (including but not limited to benchmarking with existing tools and sustainability) must be addressed also.

A2.

We have now added a comparison of FUMA with existing tools. Direct benchmarking with other tools did not seem feasible (as many of the other tools do not aim to prioritize genes, but only conduct part of the analysis needed to select genes). We did however test the outcomes of FUMA in the three empirical examples against what has already been published before on genes for the three traits (BMI, Crohn's disease, Schizophrenia)

Q3. We are aware that this paper had initially been submitted as Letter to Nature Methods. At Nature Communications, the limit to the main text (Introduction, Results, Discussion) is 5000 words and there is no word limit on the Methods section. We encourage you to make better use of this word allowance so that descriptions and conclusions drawn become clearer for the reader. Please highlight all changes in the manuscript text file.

A3.

We have reformatted the paper to suit the format of Nature Communications. The main text is now 4,469 words and the Methods section is 2,222 words. All changes, including the changes in response to the reviewers' comments are in blue text.

Q4. At the same time, we ask that you ensure your manuscript complies with our editorial policies. Please ensure that the following requirements are met, and any relevant checklist is completed and uploaded as a Related Manuscript file type with the revised article.

A4.

We have changed our manuscript where necessary, conform the life sciences checklist as well as the format checklist as a related manuscript file. In addition. We have added a data availability statement under the Methods section.

Reviewer #1

The manuscript described a web-based toolset, FUMA, to annotate significant locus of genome-wide association studies (GWAS). FUMA incorporated several databases and bioinformatics softwares to develop a pipeline that help users to better analyze GWAS results and pick up likely functional locus, both in coding regions and regulatory regions; while, in the meantime, provide easy visualization tools.

The tool is needed in the field; The databases chosen for the genomic annotation is comprehensive though straightforward; The design of the website is clear and functional.

Q5.

However, the novelty of the work is a concern, since all the bioinformatics tools and databases used by FUMA are well-established and the annotation process of FUMA is standard.

A5.

We designed this tool to reduce complexity of connecting various methods and data repositories, which is a highly-complicated task. It is now possible for all users with GWAS results, such as biologists or computer scientists, to bring all information back in the same framework and easily interpret GWAS results. This does not exist and requires know-how from databases, data structures, programming languages etc. FUMA generates output within 30 minutes in most of the time, which would otherwise have taken weeks to compile, using expertise from different individuals and by using many different data sources.

In the new version of FUMA we now also incorporate chromatin interaction mapping and provide figures that combine the chromatin interaction mapping with eQTL mapping. Since this kind of data is only recently available, this adds to the novelty of FUMA.

FUMA glues the resources together and provides the whole analysis pipeline as a service.

Q6.

There is room for the improvement of the website. The regional plots that align the annotation of multiple databases, such as CADD, regulomeDB, and Chromatine states, is informative. However, it is desired that FUMA could provide clickable links that direct the users to more detailed information at the original databases. For example, regulomeDB also provide information on cell types and transcription factor names, instead of merely a score.

A6.

We would like to thank the reviewer for this suggestion. We now incorporated hyperlinks to RegulomeDB to provide more detailed information at the external website. For CADD and the 15-core chromatin state all details are incorporated in FUMA itself. Of course, links to those databases are provided under “Link” tab on the website to allow users to obtain background information on how these resources were compiled

- Change made to the web application:
  In the regional plot page (which is linked from the results page of SNP2GENE), we have added an external link to RegulomeDB website in the SNP table (which is

displayed when a user clicks a SNP in the plot). This link opens a new tab and directly connects to the RegulomeDB page of the selected SNP.

Q7.

In my test run, there are glitches on some pages where texts and figures stacked on top of each other, making it impossible to read.

A7.

We are sorry to hear this and ran more extensive tests using various browsers such as Google chrome, FireFox and Safari, and fixed glitches when needed. In the previous version, some mobile devices did not display FUMA correctly. This was due to the grid layout of bootstrap. We now solved this by adding column tags not only for full size screen but also smaller devices such as mobile phone or tablet. If the reviewer still finds figures or tables that are not displayed properly, please let us know with a screenshot and more detailed information and we will fix this asap.

Reviewer #2

Most genetic variants associated with human disease sit in non-coding regions, but the function of these regions is hard to annotate and understand, a factor that challenges modern genetic studies of disease. It is in this context that the authors create an important new tool, FUMA for functional mapping and annotation of genetic variants associated with disease. They use this in a very striking proof of principle to identify new loci for BMI using published GWAS. Overall, I expect that this will be a widely referenced and highly cited paper, both for the new biology and for the tool. Most of my suggestions for improvement should therefore be considered minor, since this is an exciting advance that will substantially augment current genetic analyses.

The tool has two components, SNP to gene and gene to function, which are the key steps in moving from genetic association to some functional insight. I discuss each of these steps within this software and its application to BMI in turn. The SNP to gene mapping divides LEAD SNPs into those that are clearly functional within a gene, and those that have no clear function, which are than annotated based on eQTL data.

FUMA can calculate LEAD SNPs based itself based on certain (reasonable) assumptions, or take a list generated by the User, which is a key feature, since other methods such as CAVIAR or PAINTOR etc. may be used to define potentially causal SNP lists, and one needs flexibility in such a tool, since methods evolve so rapidly. This makes this a very flexible tool for its SNP to gene function, which will promote its use.

Moving from gene to function is a more far more involved and difficult problem. ANNOVAR is used to annotate SNPS, as well as CADD, Regulome DB and HMM based chromatin state from ENCODE. Non-coding or otherwise non-annotated SNPs are than matched with eQTLs from user defined tissues of interest, which is very useful.

Q8.

It is not clear if there are multiple eQTL data sets available, how they are merged, or reconciled if they give different or conflicting information. This can be easily addressed in the supplemental material.

A8.

We apologize this was unclear. FUMA includes 4 eQTL datasets. As pointed out by the reviewer, a SNP could have multiple eQTLs from distinct data sources which might provide conflicting information. We decided to provide all information from all available eQTL datasets (currently 4 but this can be extended when more become available) and not to pre-calculate a merged P-value using predefined weights for different datasets. By doing so all information is available to users allowing maximum flexibility in determining their own weights. We have added the following text to clarify this:

- Added to the manuscript

  Supplementary Note 2

  eQTL datasets were obtained from four data repositories. From GTEx v6[1], single tissue cis-eQTL data for every tested SNP-gene association for 44 tissue types was extracted (Supplementary Table 3). From Blood eQTL browser (5,311 peripheral blood samples)[2], we obtained cis-eQTLs that were pre-filtered at FDR 0.50. From BIOS QTL browser (2,116 peripheral blood samples)[3], gene-level cis-eQTLs were obtained and were pre-filtered at FDR 0.05. From BRAINEAC (134 individuals)[4], cis-eQTLs of 10 brain regions were obtained and were pre-filtered at nominal P-value 0.05. Genes were mapped to ensembl gene ID. Tested alleles were not provided for BRAINEAC eQTLs (assigned NA).

  Since FUMA contains 4 different eQTL data sources, there might be conflicting information, such that a SNPs is found to be an eQTL for a certain gene based on information from one repository but not the other. FUMA provides the evidence from the four different repositories as is, and users can decide to select only one repository or combine evidence from different repositories.

Q9.

One weakness of the mapping SNP to gene using ANNOVAR is that it is based on distance, and both functional eQTL and physical, Hi-C data indicate that many even intragenic SNPs act at distal genes. Assignment of non-coding SNPs to the closest gene is estimated to occur about 50% of the time, so 50% of the assignments might be off. The authors should consider integrating Hi-C/ChIA-PET and other data that is available via 3D genome browser (http://promoter.bx.psu.edu/hi-c/) or one of the other visualization tools, which would bring this pipeline to be truly state of the art.

A9.

The reviewed version of FUMA actually provided two different mapping options, one is ‘positional mapping’ which uses ANNOVAR annotation and is based on physical distance, as described by the reviewer. The second mapping option (‘eQTL mapping’) however is not based on physical position but is based on cis-eQTL relations between SNPs and genes. We agree with the reviewer that mapping SNPs to genes purely based on ANNOVAR annotations is not sufficient since most of the GWAS findings fall into non-coding regions which might have regulatory elements affecting expression of genes located far from the risk loci. On top of the eQTL mapping, we now also incorporated a third gene mapping based on 3D chromatin interactions;

‘chromatin interaction mapping’. For this, we implemented Hi-C data obtained from GSE87112 which contains 21 tissue/cell types. Additionally, predicted promoter and enhancer regions for 111 tissue/cell types from Roadmap Epigenetic Projects are also annotated to interacting regions. Although we currently implemented one Hi-C data set, we will implement further publicly available data, such as ChIA-PET and Capture Hi-C, when these are available. We also allow users to upload their own, custom chromatine interaction matrices.
As this is a novel way of gene mapping, we also updated the results of FUMA for the three apllications to GWAS results (BMI, CD and SCZ), and added the followings:

- Change made to the web application
  Chromatin interaction mapping is now added to SNP2GENE process with using Hi-C data of 21 tissue/cell types from Gene Expression Omnibus; GSE87112. SNPs in one end of significantly interacting regions are mapped to genes whose promoter regions overlap with another end of the interaction. Predicted enhancer and promoter regions from Roadmap Epigenomics Project for 111 tissue/cell types are also included to annotate significant interacting regions and can be used for further filtering of SNPs and mapped genes.

- Change made to the manuscript
  1. **Results: Workflow of FUMA web application (page 5 line 82)**
     Functionally annotated SNPs are subsequently mapped to genes based on functional consequences on genes by i) physical position on the genome (*positional mapping*), ii) eQTL associations (*eQTL mapping*) and iii) 3D chromatin interactions (*chromatin interaction mapping*). Gene mapping can be controlled by setting several parameters (**Supplementary Table 2**) that allow to in- or exclude specific functional categories of SNPs (**Supplementary Fig. 1**).

     (page 6 line 100)

     iii) *Chromatin interaction mapping* is used to map SNPs to genes when there is a significant chromatin interaction between the disease-associated regions and nearby or distant genes. Chromatin interaction mapping can involve long-range interactions as it does not have a distance boundary as in eQTL mapping. FUMA currently contains Hi-C data of 14 tissue types and 7 cell lines from the study of Schmitt *et al.*[11], yet new chromatin interaction data will be added when it becomes available and FUMA also allows users to upload their own chromatin interaction matrices, which is not limited to Hi-C, but also accommodates ChIA-PET, 5C or Capture Hi-C data (Methods and **Supplementary Note 3**). Since chromatin interactions are often defined in a certain resolution (as a genomic region), such as 40kb, an interacting region may span multiple genes. To further prioritize candidate genes from *chromatin interaction mapping*, information on tissue/cell type specific enhancer and promoter regions from the Roadmap Epigenomics Project[10] can be optionally integrated with interacting regions to filters SNPs and target genes (see Methods for details).

  2. **Results: Application to BMI GWAS (page 10 line 201; text updated to incorporate chromatin interaction results)**

To validate the utility of FUMA, we applied it to summary statistics of the most recent GWAS for Body Mass Index (BMI; 236,231 individuals)[43]. FUMA identified 95 lead SNPs (from 223 independent significant SNPs) across 77 genomic risk loci (Fig. 2 and **Supplementary Data 1-3**), in accordance with the original study. We first conducted positional mapping of deleterious coding SNPs and eQTL mapping (Methods) which prioritized 151 unique genes; 23 genes with deleterious coding SNPs (positional mapping) and 144 genes with eQTLs that potentially alter expression of these genes (eQTL mapping) including 16 genes that had both deleterious coding SNPs and eQTLs (**Supplementary Data 4**). The 151 genes consist of 55 genes that were also reported in the original study[43] and 96 novel genes implicated by FUMA, including 50 genes which are located outside the risk loci. These novel candidates have shared biological functions with the 55 previously known candidate genes such as ‘metabolism of carbohydrate’, ‘metabolism of lipid and lipoprotein’, ‘immune system’ and ‘calcium signalling’ (**Supplementary Data 5**). In addition, FUMA results showed that, although several genomic loci for BMI included multiple prioritized genes, a single gene was prioritized in 22 out of 43 loci which contain at least one prioritized gene (**Supplementary Fig. 2**), suggesting that these 22 genes have a high probability of being the causal gene in that region. The 22 ‘highly likely causal genes’ include several well-known genes for BMI such as *NEGR1*, *TOMM40* and *TMEM18*. The strongest GWAS association signal for BMI was on 16q.12.2 where 3 genes were prioritized; *FTO*, *RBL2* and *IRX3* (Fig. 3). These three genes were only prioritized by eQTL mapping as the positional mapping showed no deleterious coding SNPs located in these genes. The original study[43] only mentioned *FTO*, because the associated SNPs were located in this gene, however none of the associated SNPs have a potential direct affect such as coding SNPs on *FTO*. Two of the genes prioritized by FUMA (*RBL2* and *IRX3*) are physically located outside the genomic locus and are missed when using conventional approaches that prioritize genes located in the locus of interest based on LD around the top SNP. Although the *IRX3* gene was not reported in the original study[43], recent functional work has indeed validated this as the causal gene whose expression is affected by SNPs in the 16q.12.2 locus[44].

We then performed chromatin interaction mapping using Hi-C data of 14 tissue types (Methods). FUMA prioritized 208 genes (**Supplementary Data 4**), of which 39 genes are overlapped with the genes prioritized by positional and/or eQTL mappings and 165 genes are located outside of the genomic risk loci (Fig. 2). That resulted in total of 320 prioritized genes by combining three mapping strategies including 256 novel candidates which were not reported in the original study (Table 2 and **Supplementary Data 4**). These novel candidates further supported shared biological functions with previously reported known genes, such as lipid and lipoprotein metabolism, homeostatic process and various metabolic pathways, with greater number of genes compared to the mappings without Hi-C data (**Supplementary Data 5**). Out of 320 prioritized genes, 39 genes are mapped by both eQTLs and chromatin interactions including *IRX3* on the 16q.12.2 locus (Fig. 4), which further support the hypothesis that these genes are involved in the risk of BMI. From the 48 loci that contained at least one prioritized gene from positional and eQTL mappings, chromatin interaction mapping identified candidate genes in additional 13 loci (**Supplementary Fig. 2**), including loci mapped to known genes associated with BMI such as *MC4R*, *FOXO3* and *ADCY9*. The 320 prioritized genes showed enrichment in 10 GO terms, such as ‘response

to zinc ion' and 'oligopeptide binding' overlapping with multiple Metallothioneins and Glutathione S-Transferase genes whose association with obesity risk has been reported[45,46] (**Supplementary Data 6**).

3. **Results: Application to CD GWAS (page 12 line 249; text updated to incorporate chromatin interaction results)**

   To further illustrate its utility, we applied FUMA to the summary statistics of Crohn's disease[47] (CD; 6,333 cases and 15,056 controls). With FUMA, 95 lead SNPs from 184 independent significant SNPs across 71 genomic loci were identified for CD (**Supplementary Fig. 3** and **Supplementary Data 7-10**). First describing the results of positional mapping of deleterious coding SNPs and eQTL mapping, FUMA prioritized 95 unique genes from 32 loci (**Supplementary Fig. 4**), of which 39 genes were implicated by deleterious coding SNPs and 69 were implicated by eQTLs influencing expression of these genes (12 genes had both deleterious coding SNPs and eQTLs; Table 2 and **Supplementary Data 11**). The prioritized 95 genes include 37 known candidates genes that were also reported in the original study[47] including well-known CD related genes such as *NOD2*, *IL23R* and *SLC22A5*, while 58 genes were novel (**Supplementary Fig. 3**; see **Supplementary Note 4** and **Supplementary Fig. 5-7** for detail results). These novel candidates include 18 genes that are physically located *outside* the GWAS risk loci, and the novel candidates mainly share immune system related biological functions with 37 previously known genes (**Supplementary Data 12**). Chromatin interaction mapping using Hi-C data in Small Bowel and Liver prioritized 190 genes of which 17 genes are overlapped with genes prioritized by positional and/or eQTL mappings and 152 genes are located outside of the genomic risk loci (**Supplementary Data 11**). That resulted in total of 268 prioritized genes including 208 novel candidates which were not reported in the original study (Table 2 and **Supplementary Fig. 3**). From the 32 loci which are mapped to at least one gene by positional and eQTL mappings, additional 22 loci are mapped to candidate genes by chromatin interaction mapping, in which several of genes prioritized from those loci are involved in immune system and cytokine signalling pathways (**Supplementary Fig. 4** and **Supplemental Data 12**). One of these 22 risk loci, the 17q12 locus is mapped to 6 chemokine ligands by Hi-C in Liver; *CCL1*, *CCL2*, *CCL7*, *CCL8*, *CCL11* and *CCL13*. Additionally, prioritised genes include 11 cytokines (*IL4*, *IL5*, *IL10*, *IL19*, *IL23R*, *IL24*, *IL27*, *IL33*, *IL1RL1*, *IL18R1* and *IL18RAP*) wherein *IL18R1* and *IL18RAP* are also mapped by eQTLs in whole blood and, *IL23R* and *IL27* are also mapped by deleterious coding SNPs which further support the involvement of these cytokine genes in CD. Role of these chemokines and cytokines in inflammatory disease has been widely studied[48] and yet, chromatin interaction mapping identified additional relevant candidates from the risk loci. The prioritized 268 genes showed enrichment in 112 canonical pathways such as immune system and cytokine related pathways, which are known to be highly relevant to CD[49] (**Supplementary Data 13**).

4. **Results: Application to SCZ GWAS (page 14 line 284; text updated to incorporate chromatin interaction results)**

   We also applied FUMA to the most recent Schizophrenia (SCZ; 36,989 cases and 113,075 controls) GWAS summary statistics[3], and 128 lead SNPs from 269 independent significant SNPs across 109 genomic loci were identified

(**Supplementary Note 5**, **Supplementary Fig. 8** and **Supplementary Data 14-17**). Positional mapping of deleterious coding SNPs and eQTL mapping prioritized 84 unique genes, of which 36 genes were implicated by deleterious coding SNPs and 65 were implicated by eQTLs influencing expression of these genes (6 genes had both deleterious coding SNPs and eQTLs; **Supplementary Data 18**). The prioritized 84 genes include 65 genes which were previously reported as candidates in the original study[3], while 19 genes were novel (Table 2) including 14 genes which are physically located *outside* the GWAS risk loci. These 19 novel candidates have several shared biological functions with 65 previously known genes, such as 'matrisome' and 'neuronal system' (**Supplementary Data 19**). Out of 84 prioritized genes, 60 of them were also identified by the recent TWAS[50] and Hi-C[51] studies including 10 genes which are physically located *outside* the risk loci. The prioritized genes cover 34 genomic loci out of 109 of which 20 loci are mapped to single prioritized gene (**Supplementary Fig. 9**; see **Supplementary Note 5** and **Supplementary Fig. 10** for detailed results). These 20 genes are highly likely to drive the association signal in the genomic loci. Chromatin interaction mapping using Hi-C data in hippocampus and prefrontal cortex prioritized 6 genes in which *DPYD* is also mapped by a deleterious coding SNP and *VPS45* is also mapped by eQTL in frontal cortex (**Supplementary Data 18**). Out of these 6 genes, 4 of them are located outside of the genomic risk loci. Together with positional and eQTL mapping, it resulted in total of 88 candidate genes including 21 novel candidates which are not reported in the original study (Table 2 and **Supplementary Fig. 8**). The 4 genes prioritized only by chromatin interactions have shared functions with other genes such as 'regulation of response to stress' (*RWDD3*), 'intracellular signal transaction' (*SGSM3*) and several functions involved in regulation of transcriptions (*OTUD7B* and *ZBTB18*; **Supplementary Data 19**).
Enrichment was seen in several brain systems related pathways, such as Nicotinic acetylcholine receptors (nAChR), transmission across chemical synapses and long-term potentiation (**Supplementary Data 20**). nAChR is an important neuron receptor in which one of the subunits alpha-7 (*CHRNA7*) has been recently studied as a new Schizophrenia drug target[52,53]. nAChR was also identified as enriched pathways in the recent study using Hi-C in human cerebral cortex[51] that suggests potential involvement of nAChR pathway in SCZ risk.

5. **Discussions (page 15 line 334)**

The availability of biological resources that can aid in the interpretation of GWAS results, such as Hi-C and ChIA-PET, have dramatically increased recently and several studies have identified novel candidates from GWAS risk loci by integrating their results for example with chromatin interactions[51,54–57]. These technologies have the potential to identify distal interactions of promoters and enhancers. Especially for risk loci where it has been difficult to identify target genes due to the presence of gene desserts, distal interactions might point to causal gene. Indeed, we identified additional putative causal genes by performing chromatin interaction mapping on outcomes from three GWAS studies (BMI, CD, and SCZ) and the additionally identified genes based on chromatin interaction information were mostly located outside of the risk loci, and were shown to have shared function with known candidates. Although chromatin interactions are highly tissue/cell type specific, as well as time-dependent, and currently available data is still limited in those aspects, FUMA provides an option to upload custom

interaction matrices. Additionally, FUMA is built in such a way that newly published data including 3D chromatin interactions, eQTLs and other variant annotations can easily be included in the *SNP2GENE* process. That makes FUMA is a flexible web tool which can be utilized not only for new GWAS results but also for previously published GWAS to re-annotate risk loci with the latest biological data sources.

6. **Figure 1 (main text)**
   Added chromatin interaction mapping.

7. **Figure 4 (main text)**

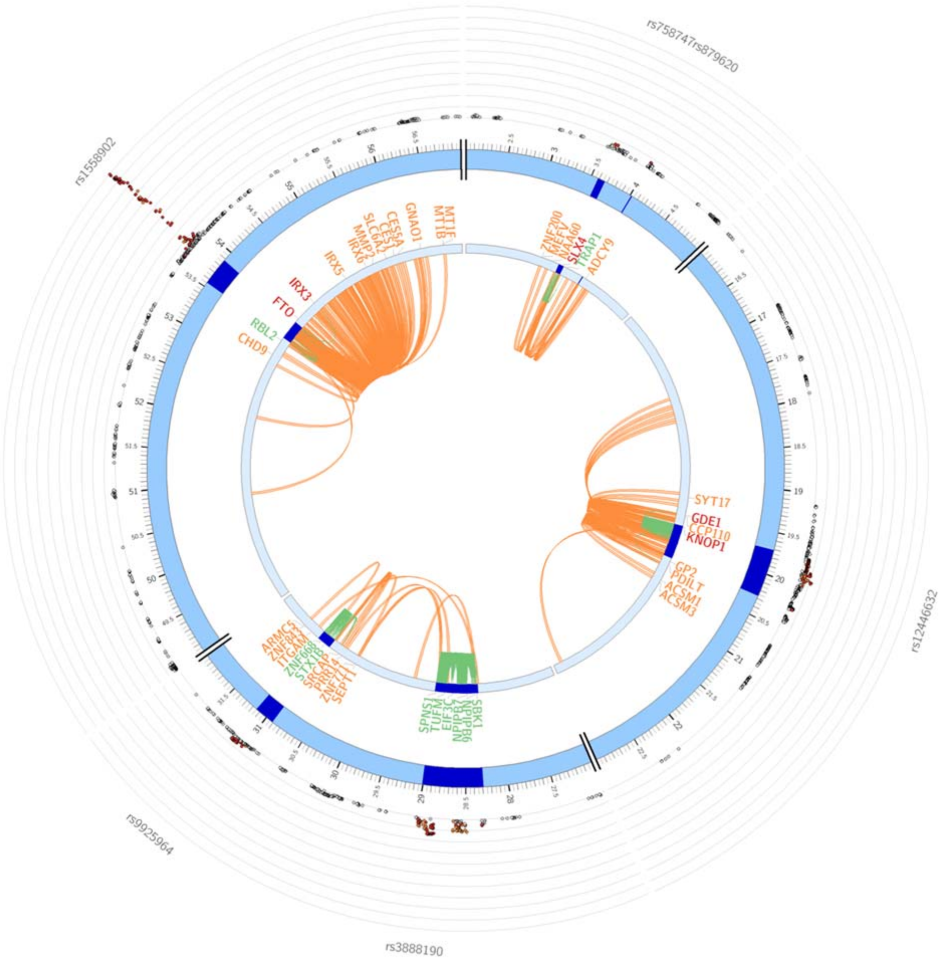

The most outer layer is the Manhattan plot displaying SNPs with P-value < 0.05. Candidate SNPs are colored based on the highest r2 to one of the independent significant loci (red: r2 > 0.8, orange: r2 > 0.6). Other SNPs are colored in grey. rsID of top SNPs per locus are labelled. The outer circle is the chromosome coordinate and genomic risk loci are highlighted in blue. Genes mapped by either Hi-C or eQTLs are shown on the inner circle. Genes mapped by Hi-C, eQTLs are colored orange and green, respectively. Genes mapped by both are colored red. Chromatin interaction and eQTLs are shown as links colored orange and green respectively.

8. **Methods: Data Pre-process (page 17 line 367)**
   Pre-processed Hi-C data for 14 tissue types and 7 cell lines were obtained from GSE87112[11] (**Supplementary Note 3**). Predicted enhancer and promoter regions for 111 epigenomes were obtained from the Roadmap Epigenomics Projects[10].

9. **Methods: Gene Mapping (page 19 line 422)**
   Chromatin interaction mapping is performed by overlapping independent significant SNPs and SNPs in LD of them with one end of significantly interacting regions in user-selected tissue/cell types. These SNPs are then mapped to genes whose promoter regions (250bp up- and 500bp down-stream of TSS by default) are overlapped with another end of the significant interactions. Optionally SNPs can be filtered for those overlapping with predicted enhancer regions of the user selected epigenomes. Similarly, mapped genes can also be filtered for having promoter regions overlap with predicted promoter regions of the user selected epigenomes.

10. **Supplementary Figure 1**
    Added chromatin interaction mapping.

11. **Supplementary Table 1**
    Hi-C data sources and predicted enhancer/promoter regions from Roadmap are added.

12. **Supplementary Table 2**
    Options for chromatin interaction mapping are added.

13. **Supplementary Note 3**
    Built-in Hi-C data of 14 tissue and 7 cell types were obtained from GSE87112[5] in which the raw data was processed to intra chromosomal interactions at 40kb resolution. We used Fit-Hi-C output which computed the significance of interactions within binned genomic regions. As suggested by Schmitt et al., interactions are filtered at FDR 1e-6 by default. However, interactions significant at FDR 0.05 are also available in FUMA and can be obtained by modifying this parameter when submitting a job.
    As an option, users can upload custom chromatin interaction matrices not limited to Hi-C but also 5C, ChIA-PET and Capture Hi-C. The input file for this is required to have the following 7 columns: (1) chromosome of region 1, (2) start position of region 1, (3) end position of region 1, (4) chromosome of region 2, (5) start position of region 2, (6) end position of region 2 and (7) parameter of significance of interaction such as FDR in which the order of region 1 and 2 are arbitral. Therefore, in the chromatin interaction mapping, the direction of interaction is not considered.

Q10.

There should at least be a way to load a 2D matrix of chromatin contacts defined by Hi-C or refined by CTCF binding sites combined with other computational predictions, etc (e.g. PMID: 27064255). Since Supplemental figure 1 has a box that says “extract all information possible”, I suggest that the authors provide a means to incorporate

or extract enhancer – promotor interactions from either Hi-C data, or computational predictions (PMID: 27064255), ATAC-seq correlations, or all of the above. Again, this would mean adding a SNP to gene annotation step that could accommodate a 2D interaction matrix, adding flexibility similar to the ability to load in a SNP list in the beginning of the process.

A10.

This is a nice suggestion, we implemented an option to upload custom chromatin interaction matrices, such as for HiC/ChiA-PET data, in the *SNP2GENE* process.

- Change made to the web application
  We have added an option to upload custom chromatin interaction matrices with a certain format which is explained in detail in the tutorial on the web application. Files can be uploaded in when a SNP2GENE job is submitted.

Q11.

Similarly, CADD has not proven that powerful for identifying missense mutations in several disorders, even though it was originally published in Nature Genetics (the current FUMA software is likely to be much more useful!). Annotation with other tools that incorporate mutational frequency and locus tolerance to damage (which seems more promising) would be useful (PMID: 26332131; PMID: 25086666), but since this field is rapidly evolving, making sure that other emerging forms of functional annotation as to the likely deleteriousness of the mutation, both non-coding and coding, can be added would be very important. If the software is available as open source, that would also aid such additions and building on to this tool in the future.

A11.

We agree that it is crucial that FUMA allows to quickly incorporate data from novel tools and resources. We have written the code of the FUMA backend in such a way that adding additional scores or functional information at both SNP and gene levels can be easily done and is partly automated. Source code of FUMA web application is available on GitHub (https://github.com/Kyoko-wtnb/FUMA-webapp) with MIT licence which allow users to contribute the improvement of FUMA application.
As pointed out by the reviewer, CADD scores provide information at the SNP level, yet scores at gene level such as intolerance to deleterious mutations or non-coding sequence stretches provides additional information to prioritized genes.
In one of the papers the reviewer suggested, i.e. Samocha et al. (2014; PMID:25086666), a statistical framework is proposed to compute excess of *de novo* mutation per gene. FUMA currently works best for common variant annotation, but we will work on incorporating de nove / very rare variants as well. We did add pLI (probability of being loss-of-function intolerance) introduced by Lek et al. (2016; PMID:27535533), as well as a gene score for tolerance of non-coding mutation called ncRVIS (non-coding residual variation intolerance score) Petrovski et al. (2015; PMID:26332131) introduced.

- Added to the web application
  Two additional gene score, pLI and ncRVIS are annotated to prioritized gene in *SNP2GENE* process. The scores are available in the “Mapped gene” table and

downloadable “genes.txt” file.

- Added to the manuscript
    1. **Methods: Gene mapping (page 20 line 436)**
    For mapped genes, two scores of intolerance to functional mutations are annotated; probability of being loss-of-function intolerant (pLI)[58] and non-coding residual variation intolerance score (ncRVIS)[59].

    2. **Supplementary Table 1**
    Data sources of pLI and ncRVIS are added

The authors subsequently use this tool to re-annotate BMI GWAS, which adds substantial new biology and demonstrates the power of this tool by identifying 96 new putative candidate genes missed in the original study and 22 loci implicating single genes, several of which are new. While individually these individual genes need additional evidence, as a group, they fall into the same functional categories as the original GWAS identified, providing confidence that this FUMA is adding substantial value. Further, the demonstration of an eQTL locus overlapping a particular risk locus, provides a demonstration of the functionality of the locus, as long as the same SNP is implicated.

Q12.

In this regard, it would be useful to know whether direct overlap between the two is needed (same SNP or surrogate in both), or whether it is locus overlap, which is slightly less convincing.

A12.

We understand from the reviewer’s question that it is unclear whether the eQTLs used for gene mapping in FUMA are matched with SNPs that are independent significant SNPs and SNPs which are in LD of them, or are merely overlapping with genomic risk loci. In FUMA, we only use SNPs that are in LD of one of the “independent significant SNPs” which are the SNPs that reached the user defined genome wide significant (5e-8 by default) and are independent of each other at the user defined r^2 (0.6 by default), to match with eQTLs in user selected databases. Therefore, the former is correct. We clarified this in the Method section.

- Change made to the manuscript
Methods: Annotation of candidate SNPs in genomic risk loci (page 18 line 400)
eQTLs are also extracted by matching chromosome, position and alleles of all independent significant SNPs and SNPs which are in LD with one of the independent significant SNPs for each user selected tissue types, wherein SNPs can have multiple eQTLs for distinct genes and tissue types (**Supplementary Note 2**).

Q13.

With regards to these applications, at least a summary in the main manuscript rather than only supplemental – such as the number of new loci and pathways in each (in a figure or table etc) would enhance the manuscript. A key question with regards to the SCZ analysis is how it is supported by recent Hi-C data (PMID:27760116 and TWAS (doi: https://doi.org/10.1101/067355). Both the TWAS and Hi-C identify many genes outside the LD block that defines these SCZ loci.

The authors also identify ACH signaling as a pathway, which is interesting, and was also identified in the Hi-C work – that could be emphasized and cited as it is an external validation of this current work, as long as the loci actually overlap.

A13.

As suggested by the reviewer, we have moved the summary of the applications (CD and SCZ GWAS) to the main text. We now also added results with chromatin interaction mapping using Hi-C data for the three applications (BMI, CD, SCZ) and compared the results of the SCZ GWAS with recent TWAS and Hi-C studies in the Result section. The changes made to the manuscript regarding this are answered in A9. We also added Table 2 in the main text as suggested by the reviewer.

- Added to the manuscript
  Table 2 (main text)

GWAS	Risk loci	Reported genes in the original study	positional mapping	eQTL mapping	chromatin interaction mapping	Total*	Genes located outside the risk loci	novel candidates	Loci contain prioritized genes
BMI	77	117	23	144	208	320	204	256	61
CD	71	115	39	69	190	268	152	208	54
SCZ	109	349	36	54	6	88	19	22	38

*The number of unique genes mapped by one of the positional, eQTL and chromatin interaction mappings

Reviewer #3

The web service (FUMA) presented by Watanabe et al combines a number of typical steps frequently taken in the analysis of GWAS data. They have illustrated their framework at the example of three representative data set (BMI, CD, SCZ) to produce priority lists and new suggestions for “causal” genes.

Q14.

Novelty.
The work includes suggestions for new genes that may be of interest for specialists working on the respective diseases. But none of the new genes were validated or evaluated beyond a cursory literature screen.

A14.

FUMA is an in-silico tool that aims to provide rapid and extensive information to facilitate gene prioritization. Validation of prioritized genes requires functional experiments, and is beyond the scope of the current work. We hope that FUMA aids in selecting the most likely causal genes for further (labour intensive and expensive) functional follow-up.

Q15.

Also for the computational aspects, I could not identify any novel methodological developments or components.

A15.

FUMA aims to facilitate post-GWAS annotation and prioritization. The current version includes chromatin interaction mapping, which is a novel way of mapping SNPs to genes and interpreting GWAS results. See our more detailed response in A5.

Q16.

Popular tools and data resources have been combined. While I recognise the importance of such efforts, it is a major omission that prior works in the field of GWAS analysis have not been cited. There is even an old review (Hou & Zhao, Front Genet. 2013) that includes many tools and web services for GWAS prioritisation.

A16.

We apologize for this omission and thank the reviewer for pointing this out, we have now added a comparison of FUMA with multiple bioinformatics tools and data sources and cited the reference mentioned by the reviewer (see A17).

Q17.

A thorough comparison with such works is necessary to assess the usefulness of this new web server.

A17.

As suggested we have added a comparison with other tools that perform post-GWAS analyses. A systematic comparison with FUMA is performed by feature comparison of widely used bioinformatics tools and databases in post-GWAS follow-up analyses. Note that comparison of the outcome of the tools is not feasible as the purpose of each is different and input/output formats are not comparable.

- Added to manuscript

1. **Results: FUMA covers various features of existing tools (page 8 line 152)**
   As a variety of bioinformatics tools have been developed to obtain insights in GWAS results[23–25], we compared the list of features available in FUMA with the features available in other tools, and describe these further below (and see Table 1).
   LD calculation is the first step to characterize risk loci of GWAS by computing population specific LD structure, so called clumping which identifies independent significant SNPs and defines the genomic risk loci. PLINK[26] is the most widely used software for this task which takes GWAS summary statistics (requiring a reference panel) or genotype data as input. In FUMA, this task is automated by using pairwise LD (r^2) of SNPs in the reference panel (1000 genomes project phase 3[27]) pre-computed by PLINK, resulting in a list of independent significant SNPs, lead SNPs and genomic risk loci based on the GWAS input file. FUMA also adds SNPs to the identified risk loci that do not have a P-value (i.e. they were not available in the GWAS input file), but that are LD proxies of the identified lead SNPs, as these SNPs might be causally relevant. Alternatively, users can pre-compute lead SNPs or risk loci and upload these to FUMA.
   Variant Annotation is required to obtain information on biological consequences of SNPs in the risk loci. There are several tools such as ANNOVAR[12] and VEP[28] which annotate functional consequences on genes, and variant scores such as deleteriousness and phylogenetic conservations (extensive review is available in Hou and Zhang[29]). Particularly for non-coding SNPs, SCAN[30], RegulomeDB[14] and HaploReg[31] annotate regulatory information, such as eQTLs,

enhancer/promoter regions and transcription factor binding sites (TFBS) (see Tak and Farnham[32] for extensive overview). Although SCAN and HaploReg correct for LD, the input of the tools mentioned above is a list of SNPs of interest which does not take genetic associations into account and thus requires pre-processing of GWAS results by the user. FUMA performs annotation of SNPs that are in LD of independent significant SNPs in a single flow, and does not require additional data preformatting.

Gene-based test / Gene-set analyses are methods that enable to summarize SNP associations at the gene level and associate the set of genes to biological pathways. For instance, VEGAS performs permutation based simulation[33,34], MAGMA employs multiple linear regression[35] and Pascal computes sum and maximum of chi-squared statistics[36] to obtain gene-based P-values. Additionally, there are several tools that perform not only gene-based test but also gene-set analyses using full distribution of genetic associations (e.g. MAGMA[35], MAGENTA[37], INRICH[38] and DEPICT[39]). FUMA implements MAGMA gene-based analysis and gene-set analysis on the full GWAS input data. In addition, genes prioritized by SNP2GENE or by the user are also tested for overrepresentation in various gene sets in *GENE2FUNC* process.

Visualization is one of the essential features that allows (quick) insights into the GWAS results, e.g. summarizing annotated information of SNPs and genes. LocusZoom is one of the most widely used visualization tool for GWAS results which plots LD structure of a risk locus, gene locations as well as SNP association values[40]. LocusTrack is an extension of LocusZoom which also plots additional information together such as Chip-seq and chromatin state[41]. 3D Genome Browser is a recently developed web application which contains comprehensive 3D chromatin interaction datasets such as Hi-C and ChIA-PET[42], though it does not integrate with GWAS summary statistics. These tools are primary focused on visualization of a subset of functionally relevant data sources. FUMA integrates results from multiple lines of evidence and provides interactive visualization of results, facilitating rapid interpretation.

The current lack of a single platform that integrates all possible resources for post-GWAS annotation hampers our understanding of GWAS results, as different GWAS studies may use a different selection of queried resources rendering their post-GWAS interpretation incomplete and difficult to compare. FUMA provides a central place for a wide variety of post-GWAS annotation strategies and to our knowledge is the most versatile tool in doing so.

2. Table 1 (main text)

Tools	Format	GWAS summary statistics	LD	Functional consequences on genes	Regulatory elements	eQTLs	3D chromatin interactions	Prioritize SNPs	Map SNPs to genes	Gene expression	Pathways and gene sets	Prioritize genes	Visualization
**LD calculation**													
PLINK	St	x	x										
**Variant Annotations**													
ANNOVAR	St			x	x			x	x				
VEP	St			x	x			x	x				
SCAN	Web		x			x		x		x			
ReglomeDB	Web				x	x		x					x
HaploReg	Web		x		x	x		x					
**Gene-based test / Gene-set analyses**													
VEGAS	St	x							x			x	
MAGMA	St	x							x		x	x	
Pascal	St	x							x		x	x	
MASENTA	St	x							x		x	x	
INRICH	St	x							x		x		
DEPICT	St	x							x		x	x	
**Visualization tools**													
LocusZoom	St/Web	x											x
LocusTrack	St/Web	x			x								x
3D genome browser	Web						x						x
**FUMA**	Web	x	x	x	x	x	x	x	x	x	x	x	x

St: Standalone software, Web: Web based application.

Q18.

Accessibility.
In general it would be preferable to use their service without registration, and to offer login (and perhaps an analysis history) only as an option for recurring users.

A18.

We now offer built-in examples without registration. This allows users to browse through the example results, and see the functionality of FUMA. For security reasons, we do require any user that wants to upload GWAS summary statistics to register.

- Added to the web application
  From the "Browse Examples" tab on top of the page, we have prepared three example results which does not require to login/register to browse. The result page contains full functionality of FUMA which allow users to use interactive plots and download results.

Q19.

When I had tried to login as example4@fuma.nl I got an error (see attached file). Therefore I could not evaluate the web service other than through the descriptions in the manuscript.

A19.

We are very sorry this happened. We now tested FUMA on 3 different browsers (Chrome, Safari and Firefox) and on separately on Windows 7, Windows 10, Mac OSX, Linux distribution (Ubuntu and Mint) using the example4 login and were unable to re-produce the error. We hope the issue is solved, but if it happens again, please let us know and we fix it.

Q20.

As is common practice for larger collection of data and annotations, it would also be useful to have an API for more programmatic access and batch processing.

A20.

Developing API is a good suggestion since some users may prefer scripts or command line functions to a web interface. However, creating an API is not trivial and will take some time. We would like to make the current version of FUMA available in the community and are working with other groups, such as the Psychiatric Genomics Consortium, to create API and include FUMA in a command-line based post-GWAS annotation pipeline.

Q21.

Sustainability.
Undoubtedly extensive efforts went into collecting and preparing the various data sources for functional annotation of SNPs. Therefore it would be important to understand if, and to what extent, this process has been automated. Otherwise the service will soon be outdated and unmaintainable by the onslaught of new data and changing annotations. It would be equally important to describe in more detail the infrastructure and computational resources which are in place to sustain this service long-term.

A21.

We agree with the reviewer that sustainability is one of the most important aspects for this kind of bioinformatics tool since a variety of new methods and databases are constantly evolving. Updating tools and database are partially automated, e.g. once new data is available, this can be easily pre-processed the format and added to FUMA. We currently have one dedicated person for the next 3 years working to make sure FUMA remains updated and to prepare new datasets (with new formats and new requirements for data normalization), and after this period aim to continuously have a researcher part-time available for this project. In addition, we are setting up an advisory board for FUMA consisting of experts in the field of genetics, and have opened a google forum (https://groups.google.com/forum/#!forum/fuma-gwas-usersn) on which users can not only report bugs or errors but also suggestions of data/tools to be implemented into FUMA as well as open contributions on GitHub.

Q22.

Data formats
Throughout the manuscript the data formats were unclear or imprecise. I assume that the input "GWAS summary statistics" refers to VCF files, but which version? Similarly, also the output formats and standards should be defined more carefully.

A22.

We apologize this was unclear. The tutorial section (SNP2GENE/input files) on the FUMA website provides instructions on input formats. We have now added additional information in the Supplementary Note about input and output formats. The input GWAS summary statistics is a plain text file with multiple columns, and needs to contain at least rsID or both chromosome and position, and GWAS P-value.

- Added to the manuscript

  Supplementary Note 1

  As input, FUMA takes GWAS summary statistics as a plain text file. Since there are multiple applications which are widely used to perform GWAS such as PLINK, SNPTEST and METAL, to minimize input data formatting, FUMA automatically captures headers of the output files from these three tools. Users can also provide custom header names instead. Tab and single or multiple white spaces are accepted as delimiters.

  The downloadable output files from both *SNP2GENE* and *GENE2FUNC* processes are tab delimited plain text files. The descriptions of each file are below.

  GenomicRiskLoci.txt

  Genomic risk loci defined by independent lead SNPs and maximum distance between their LD block.

    - Genomic locus : Index of genomic rick loci.
    - uniqID : Unique ID of SNPs consists of chr:position:allele1:allele2 where alleles are alphabetically ordered.

      ...

  (see Supplementary Note 1 for complete list of output format)

Reviewer #1 (Remarks to the Author):

The authors have addressed my comments. The revised manuscript and the website have been significantly improved. Therefore, I recommend the publication of this work on Nature Communication. I hope the authors will continue making progress and provide outstanding services to the community.

Reviewer #2 (Remarks to the Author):

The authors have done an excellent job replying to all of the reviews, mine and the others. They have added all of the tools suggested and made the FUMA more flexible -- they are providing a person dedicated to continuing updates for 3 years. This will make this a widely used tool.

Reviewer #3 (Remarks to the Author):

The authors have significantly improved the manuscript. Especially the inclusion of HiC data is an important and novel addition over the previous version. At the same time the authors have also demonstrated much clearer where the FUMA web service goes beyond previous efforts (Table 1) and cited them properly. I would encourage the authors to state their cookies policies more explicitly. This had caused significant problems and delays during the revision.
